# Genetically engineered human cell–based microrobots for selective cancer cell death

**DOI:** 10.1126/sciadv.aea9831

**Published:** 2026-04-29

**Authors:** Nihal Olcay Dogan, Eylül Suadiye, Julia Unangst, Cem Balda Dayan, Gunther Richter, Ahmet Cingöz, Tugba Bagci-Onder, Metin Sitti

**Affiliations:** ^1^ Physical Intelligence Department, Max Planck Institute for Intelligent Systems, 70569 Stuttgart, Germany.; ^2^ Institute for Biomedical Engineering, ETH Zurich, 8092 Zurich, Switzerland.; ^3^ Materials Central Scientific Facility, Max Planck Institute for Intelligent Systems, 70569 Stuttgart, Germany.; ^4^ Robotic Materials Department, Max Planck Institute for Intelligent Systems, 70569 Stuttgart, Germany.; ^5^ School of Medicine, Koç University, 34450 Istanbul, Turkey.; ^6^ Beykoz Institute of Life Sciences and Biotechnology, Bezmialem Vakif University, 34820 Istanbul, Turkey.; ^7^ College of Engineering, Koç University, 34450 Istanbul, Turkey.

## Abstract

Medical microrobots have strong potential for targeted therapeutic delivery; however, current systems achieve only physical targeting, and once at the target site, they are unable to distinguish healthy cells from cancerous ones because of the lack of biological selectivity. Here, we present a biohybrid microrobot system that combines magnetic targeting with biological selectivity. The microrobots are derived from human embryonic kidney cells genetically engineered to produce tumor necrosis factor–related apoptosis-inducing ligand (TRAIL), a molecule that induces cancer cell death in multiple tumor types without damaging healthy cells. Engineered cells are then conjugated to biocompatible magnetic Janus particles—silica beads half-coated with FePt nanofilms—to enable external magnetic control. With magnetic fields, the microrobots accumulate around the tumor spheroids and continuously release TRAIL for several days, leading to selective cancer cell death while avoiding damage to healthy cells. This study combines microrobotics with genetically engineered cell therapies to achieve a targeted, prolonged, and cancer-selective therapeutic delivery.

## Teaser

Magnetically guided human cell–based microrobots selectively kill cancer cells without harming surrounding healthy tissues.

## INTRODUCTION

Mobile microrobots—remotely controlled active machines operating at the cellular scale—offer a promising strategy for targeted therapeutic delivery by navigating the diseased sites using external energy sources, such as magnetic, acoustic, or optical fields (*1*–*5*). In the past decade, substantial progress has been made in microrobot design, actuation performance, drug loading capacity, and real-time tracking with medical imaging modalities, further enhancing their potential for medical applications (*6*–*12*). However, despite these advances, microrobots have been mainly fabricated from synthetic materials that may induce immune responses or exhibit cytotoxicity in vivo, thereby limiting their medical functionality because of the unwanted interactions with the body’s immune system and foreign body responses (*13*–*15*). In addition, the synthetic microrobots are often constrained by limited cargo-carrying capacity when compared to living cells (*16*, *17*). In contrast, the body’s living cells can continuously synthesize and secrete bioactive molecules throughout their lifespan, offering enhanced therapeutic delivery with reduced immunogenicity (*12*, *15*, *18*–*21*). Therefore, developing biohybrid microrobots, especially from living human cells, can enable prolonged and high-level therapeutic release, improved efficacy, and innate biocompatibility, making these systems well suited for medical applications (*15*, *22*–*25*). Despite substantial progress in the field, current microrobots can achieve only physical targeting without inherent biological selectivity. Although microrobots can be localized to the target region, once they reach the target area, they cannot distinguish cancerous cells from healthy cells, highlighting the need for further exploration.

In contrast, cell therapies can achieve biological targeting rather than physical targeting by using a patient’s own cells as “living drugs,” which can proliferate as they circulate within the body while actively seeking out disease-associated cells or microenvironments to carry out their therapeutic function (*26*). However, this approach still does not inherently provide biological selectivity, as these cells may still interact with or affect nontarget healthy tissues that share similar markers or microenvironmental cues (*27*). A clinical example is tumor-infiltrating lymphocytes, in which patients’ own tumor-resident immune cells are expanded and reinfused to treat cancer without additional genetic modifications. However, the efficacy of such treatments is limited by the immunosuppressive tumor microenvironment, which compromises tumor-infiltrating lymphocyte function and enables cancer cells to escape immune-mediated killing (*28*, *29*). To address these limitations, genetic engineering has been incorporated into cell therapies to enhance their performance. Through viral transduction and nonviral transfection methods, synthetic receptors or other molecules can be expressed on cells to improve target cell recognition and cytotoxic activity while helping engineered cells remain functional despite the tumor-induced immunosuppression (*30*). As a result of these engineered receptors or molecules, cells can detect and attack cancer cells even when tumors attempt to hide. Chimeric antigen receptor (CAR) T cell therapy is one of the most successful clinically approved therapies that involves genetic engineering. In this approach, a patient’s T cells are genetically modified to express specialized receptors, called CARs, that target tumor antigens, such as CD19 and BCMA (B cell maturation antigen), enabling them to attack tumor cells even when natural immune pathways fail (*31*, *32*). Despite their great success in blood cancers, CAR T cell therapies still face some challenges: (i) Their dependence on a single antigen for recognition limits their effectiveness to tumors expressing only that specific marker, and therefore, treatment can fail if cancer cells lose or alter the antigen through antigen loss or tumor heterogeneity (*33*); (ii) many target antigens are expressed on both healthy and tumor cells, thus causing unintended toxicity to healthy cells as well (*34*); and (iii) CAR T cells require direct cell-to-cell contact to initiate cytotoxicity and, when combined with their limited penetration, further compromise their effectiveness in solid tumors (*31*, *35*). As a result of these challenges, new therapeutic approaches are needed that can induce broad-spectrum anticancer toxicity without relying on single-marker recognition or direct cell-to-cell contact for functioning.

Given that mobile microrobots provide physical targeting and genetically engineered cell therapies offer biological targeting, merging these two strategies could establish a powerful platform that combines targeted navigational capabilities with highly effective therapeutic cell functions. Although this concept has been explored in a few preliminary studies, its potential remains limited. For instance, microrobots constructed from CAR T cells and actuated via magnetic-acoustic fields have shown potential in preclinical cancer models (*24*). However, their dependence on a single tumor-specific antigen, such as CD19, limits their applicability only to CD19-expressing tumor models. Moreover, given that CD19 is expressed on both healthy and tumor B cell populations, this strategy also eliminates healthy B cells, reflecting antigen specificity rather than healthy or cancer cell specificity, which can still result in unwanted toxicity. In addition, because CAR T cells require direct physical cell-to-cell contact to trigger cytotoxicity, together with the absence of sustained therapeutic release, their overall effectiveness remains limited (*36*). In other studies, artificial helical microswimmers fabricated from synthetic materials and functionalized with plasmid DNAs have achieved targeted gene delivery at the single-cell level (*37*, *38*). Although these systems offer precise gene delivery, they lack therapeutic relevance, as their effects are limited to the single-cell level.

In this study, we introduce a strategy that integrates microrobotics with genetic engineering to achieve selective cancer cell death without harming healthy cells while enabling broad-spectrum cell death in diverse cancer cell types. For this, we genetically engineered living human embryonic kidney cells to express tumor necrosis factor–related apoptosis-inducing ligand (TRAIL), which is a selective anticancer ligand that induces cell death in multiple cancer cell types without harming healthy tissues (*39*–*41*). Mechanistically, TRAIL binds to the death receptors, death receptor 4 (DR4; also known as TRAILR1) and DR5 (also known as TRAILR2), initiating an intracellular signaling cascade that ultimately leads to programmed cell death (*42*, *43*). These receptors are often overexpressed in tumor cells, making them highly sensitive to TRAIL-induced cell death. In contrast, healthy cells generally express low or negligible levels of DR4/DR5 receptors and are therefore highly resistant (*44*). As a result of this differential expression of receptors between healthy and cancerous cells, cancer cells can be selectively eliminated without damaging healthy tissues.

After genetically modifying human embryonic kidney cells with the *TRAIL* gene, the cells were further functionalized with magnetic Janus particles, which consist of 500-nm-diameter silica beads partially coated with a 60-nm-thick biocompatible iron platinum (FePt) magnetic nanofilm. These particles bound stably to the cell membrane without impairing cell viability and function, achieving a high conjugation yield (~92.1%) and enabling magnetic actuation with torque-based surface rolling locomotion. The resulting TRAIL-expressing cell–based microrobots secreted TRAIL while navigating along assigned trajectories, selectively inducing apoptosis in multiple cancer cell types without harming healthy cells. They were magnetically navigated toward three-dimensional (3D) tumor spheroids and effectively eliminated the renal cell carcinoma tumor cells. Their ability to be magnetically guided and concentrated near tumor regions in vitro demonstrates the feasibility of localized and higher concentrations of TRAIL delivery directly to the diseased site, thereby increasing the exposure of cancer cells to TRAIL, enhancing antitumor effects while preserving healthy tissues. Unlike previous approaches, these biohybrid microrobots integrate physical targeting with biological selectivity. Physical targeting can enable controlled delivery of therapeutic cells to their intended site rather than navigating through the systemic circulation, thereby minimizing the loss of the therapeutic agents because of systemic clearance or short half-lives. Complementarily, biological selectivity can provide that once the microrobots are at the target region, therapeutic cells preferentially act on cancer cells while protecting adjacent healthy tissues, minimizing unwanted toxicity. Moreover, the modular design of this strategy allows for the easy substitution of both the therapeutic cell type and gene, making the platform highly adaptable for diverse biomedical applications.

## RESULTS

### Development of human cell–based microrobots for selective cancer cell death

An effective cancer therapy should eliminate cancer cells without harming healthy tissues—a goal that remains challenging to achieve with conventional chemotherapeutic agents, as well as currently existing cell therapies, and microrobotic approaches (*45*). As a goal of this study, we developed biohybrid microrobots derived from living human cells that selectively target and destroy cancer cells while protecting surrounding healthy tissues, thereby minimizing off-target effects. This high degree of specificity is mainly achieved through TRAIL-mediated apoptosis. Secreted TRAIL binds to the DR4/DR5 death receptors on the cell surface and initiates a signaling cascade, causing programmed cell death (*46*). On tumor cells, these receptors are usually highly expressed, making them highly sensitive to TRAIL-induced cell death. In contrast, healthy cells typically express low or negligible levels of DR4/DR5 receptors, making them highly resistant to TRAIL-induced apoptosis, thus providing a strong biological basis for selective cancer cell killing (*44*). In addition to biological selectivity, our system uses magnetic guidance as a complementary mechanism to achieve controlled movement and targeted localization of the microrobots. External magnetic fields enable precise navigation and accumulation of the microrobots near the tumor site, thereby increasing the local concentration of TRAIL around the tumor, and can help ensure that it is released predominantly in the vicinity of cancer cells, rather than dispersing passively throughout the body. Given that TRAIL is a diffusible molecule and its apoptotic activity does not require direct physical contact between the therapeutic cell and the cancer cell, effective apoptosis can still be induced even if the microrobots do not remain anchored at a specific location within the tumor (*47*). This synergistic approach can enhance both the local therapeutic concentration and functional half-life of TRAIL. As a result, TRAIL released at the target site can induce selective cancer cell death while leaving healthy tissues unharmed.

**Fig. 1.**
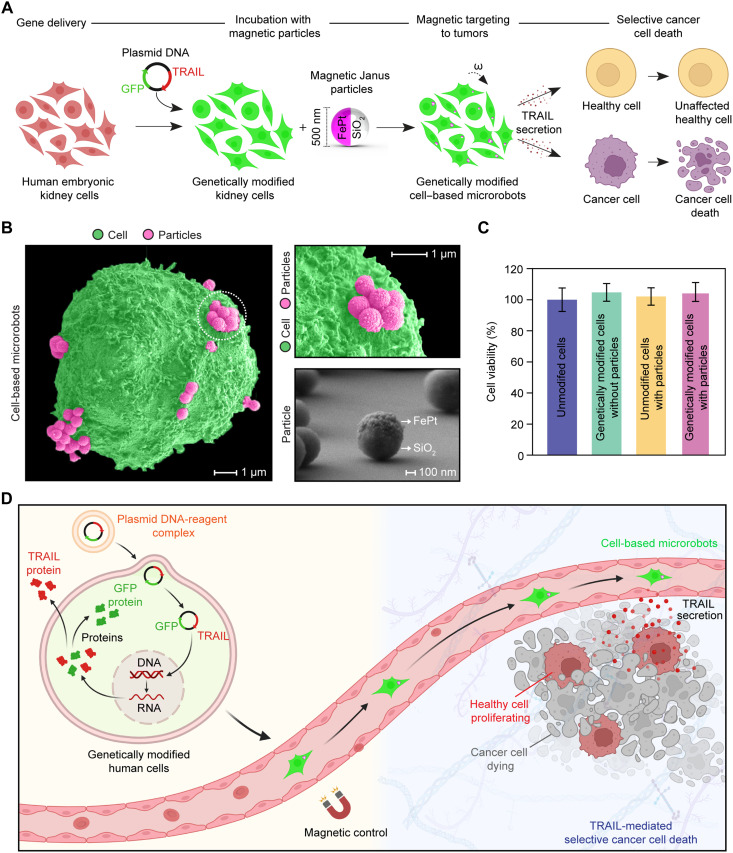
Development of human cell–based microrobots for selective cancer cell death. (**A**) Human embryonic kidney 293T cells were genetically engineered with plasmid DNA encoding two functional units: *TRAIL* for selective cancer cell death and *GFP* for fluorescence tracking. The genetically modified cells were then conjugated with magnetic Janus particles, which are 500-nm-diameter silica beads coated with a 60-nm-thick FePt magnetic nanofilm, for remote magnetic control. The resultant biohybrid microrobots, referred to as cell-based microrobots, can actively navigate toward target sites and secrete TRAIL extracellularly to induce localized cancer cell death without damaging surrounding healthy tissues. Created in BioRender. N. O. Dogan (2026), https://biorender.com/knfax2i. (**B**) SEM images showing cell-based microrobots with magnetic Janus particles attached to the cell surface, with no evidence of internalization. Images were pseudocolored. Scale bars, 1 μm. SEM image of magnetic Janus particle, confirming the deposition of FePt on its silica core. Scale bar, 100 nm. (**C**) 293T cell viability, assessed on the basis of intracellular ATP quantification, demonstrating negligible cytotoxicity after genetic modification, particle conjugation, and their combination (i.e., cell-based microrobots), confirming the high biocompatibility of the engineering process. Data are presented as the means ± SD from *n* = 4 independent biological replicates, each measured in at least three technical replicates. (**D**) Conceptual schematics depicting cell-based microrobots magnetically guided to a target tumor site, where continuous, local TRAIL secretion eliminates cancer cells without affecting nearby healthy tissues. Created in BioRender. N. O. Dogan (2026), https://biorender.com/e18h681.

Our biohybrid microrobots were constructed from living human embryonic kidney 293T cells, which were genetically modified via plasmid DNA encoding both *TRAIL* and green fluorescent protein (*GFP*). The TRAIL ligand is known for its tumor-selective cytotoxicity (*43*), while the fluorescent reporter protein GFP allows for visualization, monitoring, and rapid verification of successful gene delivery throughout the experimental workflow (Fig. 1A) (*48*). The genetically modified *TRAIL*- and *GFP*-coexpressing cells were subsequently conjugated to magnetic Janus particles, enabling a swarm of them to be controlled and navigated using external rotating magnetic fields. The magnetic Janus particles were fabricated from 500-nm-diameter silica beads, half-coated with a 60-nm-thick FePt nanofilm. We selected a 500-nm particle size because smaller particles might generate insufficient magnetic forces for effective actuation, whereas larger particles may impair cell viability or motility and are more difficult to attach to the cell membrane stably (*49*). In our study, this selected size showed no detectable adverse effects on cell viability while supporting magnetic actuation. The biocompatibility of magnetic Janus particles was evaluated at concentrations of 0.05 to 0.4 mg/ml over 24 to 72 hours, with no notable differences observed in cell viability compared to untreated controls (fig. S1A). In all groups, at least 86% of 293T cells survived, indicating minimal cytotoxicity and good tolerance of 293T cells to magnetic Janus particles (fig. S1B). Next, FePt was selected because of its strong magnetic properties (*37*), high biocompatibility (*15*), and high imaging contrast (*12*, *50*) across various medical imaging modalities, including magnetic resonance imaging, computed tomography, and optoacoustic imaging, making it an ideal material for developing magnetic microrobots. Although iron oxide nanoparticles and nickel- or cobalt-based coatings are commonly used in the microrobotics field, iron oxide exhibits relatively weak magnetic responsiveness under physiological conditions. This limitation results in insufficient torque for actuating large objects, such as whole cells (~10 to 20 μm), or leads to lower propulsion velocities compared with ferromagnetic materials (*51*). On the other hand, nickel and cobalt are cytotoxic, making them unsuitable for cell-based microrobots or in vivo applications (*52*, *53*). As a result, FePt was selected as the most suitable magnetic material for our system, as its combination of strong magnetic properties and high biocompatibility enables effective magnetic control of the particle-attached cells while preserving cell viability.

FePt coating on silica beads was first confirmed through scanning electron microscopy (SEM), which provides detailed insights into particle surface morphology (Fig. 1B). Particle surfaces were also mapped using energy-dispersive x-ray spectroscopy, which revealed homogeneous distribution with nearly equal atomic compositions of Fe [56.57 ± 1.04 atomic % (at %)] and Pt (43.43 ± 2.98 at %) (table S1 and fig. S2). Such compositional uniformity is favorable for the uniform magnetic response and chemical stability of FePt-based particles. Next, dynamic light scattering and zeta potential measurements were performed on FePt-coated silica beads. The silica cores, reported by the manufacturer to be ~400 to 600 nm in diameter with an average size of ~500 nm (confirmed by SEM; Sigma-Aldrich, cat. no. 808989), were further coated with a 60-nm-thick FePt layer. The resulting FePt-coated silica beads showed a main hydrodynamic peak at 619 ± 40 nm and a *Z*-average at 763 ± 59 nm, consistent with the expected size after FePt deposition. Zeta potentials were measured on average at −19.5 ± 0.8 mV, indicating moderate colloidal stability of the particles in culture medium (table S2 and fig. S3).

Next, the particles were incubated with human 293T cells overnight to allow membrane attachment. Unlike our previous findings with macrophages, which internalize Janus particles via phagocytosis, 293T cells retained the particles on their surface (*12*, *15*). SEM analysis confirmed that a single 293T cell could stably bind multiple Janus particles, even after repeated washing and centrifugation, with no evidence of internalization (Fig. 1B). Moreover, particles consistently showed a tendency to associate with cells rather than freely accumulating on the culture plate (fig. S4), demonstrating strong and stable cell-particle association. Although 293T cells are not phagocytic, this attachment may be supported by localized membrane deformation or partial wrapping through nonphagocytic endocytosis, contributing to the stability of the cell-particle conjugates. After confirming stable attachment under static conditions, we tested the stability of the cell-particle conjugates under physiologically relevant shear stress. Shear levels corresponding to venous shear stresses and arterial shear stresses caused no particle detachment, indicating that the cell-particle conjugates remain stable under low-to-moderate physiologically relevant shear (fig. S5, A to C). Conditioned medium from shear-exposed cell-based microrobots showed no toxicity toward healthy BJ human skin fibroblasts or human adipose-derived mesenchymal stem cells (hAMSCs), suggesting that any potentially detached particles do not induce off-target toxicity in healthy cells (fig. S5D).

As part of the safety and feasibility assessment, we evaluated the potential cytotoxic effects on 293T cells associated with genetic engineering and the conjugation of magnetic Janus particles. Transfection reagents used in genetic modification can induce cytotoxic effects, which may compromise both gene delivery efficiency and cell viability (*54*). Moreover, as genetic modification already imposes stress on cells, the additional conjugation of magnetic nanoparticles could further affect viability. To assess cell viability after each modification step, we used different methods that target multiple cellular parameters: CellTiter-Glo assay for adenosine 5′-triphosphate (ATP) quantification, 3-(4,5-dimethylthiazol-2-yl)-2,5-diphenyltetrazolium bromide (MTT) assay for mitochondrial metabolic activity, and phase-contrast microscopy for morphological evaluation. CellTiter-Glo assay measures the intracellular ATP level of cells to determine viability, given that ATP can only be synthesized by metabolically active and viable cells. As soon as the CellTiter-Glo reagent is added to the well, cells lyse and release their ATP. Then, an enzyme called luciferase catalyzes a reaction in which ATP is converted into a luminescent signal, which is directly proportional to the number of viable cells (*55*). Our CellTiter-Glo measurements indicated no cytotoxicity at any modification stage, unmodified 293T cells, genetically modified cells, unmodified cells with particles, and genetically modified cells with particles, indicating that neither genetic engineering nor particle attachment affected cell viability (Fig. 1C). A similar principle allows the MTT assay to evaluate cell viability by using the principle that living cells with metabolically active mitochondria can convert the yellow MTT substrate into purple formazan crystals through an enzymatic process. As dead cells lack this enzymatic activity, purple formazan production directly correlates with the number of viable cells (*56*). MTT assay results indicate that neither the genetic modification nor particle attachment impaired mitochondrial metabolism, as indicated by similar mitochondrial metabolic activity in all groups (fig. S6). Last, morphological changes provide key insights into cell differentiation and functional status; therefore, we examined the cells using phase-contrast microscopy, which revealed no noticeable morphological changes across unmodified 293T cells, genetically modified cells, and genetically modified cells with particles, consistent with the viability assay results (fig. S7). Collectively, these results confirm that the design strategy used in producing such microrobots is highly biocompatible. The resulting biohybrid microrobots from human cells, referred to as cell-based microrobots, demonstrate a strong response to external magnetic fields, enabling their active and directional movement toward targets.

The resulting human cell–based biohybrid microrobots can be magnetically guided and concentrated near the tumor region, where they secrete TRAIL locally rather than dispersing passively throughout the body. This localized release may contribute to more effective killing of nearby cancer cells while minimizing damage to surrounding healthy tissues (Fig. 1D). TRAIL is a diffusible protein; therefore, it does not need direct physical contact of therapeutic cells with the cancer cells to activate apoptotic pathways. Thus, even if the microrobots are not fixed at a specific point within a tumor, the secreted TRAIL can diffuse through the microenvironment to induce apoptosis in nearby cancer cells.

### Optimization and characterization of cell-based microrobots

In biological fluids, naked DNA is rapidly degraded by nucleases; therefore, a delivery system is needed to protect the DNA and facilitate its uptake by cells (*57*, *58*). Among the various methods of introducing plasmid DNA into 293T cells, we used a chemical transfection approach using a nonliposomal transfection reagent. Positive charges of the transfection reagent neutralize the negatively charged plasmid DNA and facilitate its uptake by negatively charged cell membranes (*59*). This approach was selected for its high efficiency, low cytotoxicity across various cell lines, ease of use, and lack of special equipment requirements. However, optimizing this method for each cell line is necessary to achieve maximum transfection efficiency (*58*). Therefore, we tested four different transfection reagent-to-DNA ratios according to the manufacturer’s recommendations (table S3). The genetically modified 293T cells were first characterized for the expression of GFP and TRAIL at multiple ratios and time points under particle-free conditions.

**Fig. 2.**
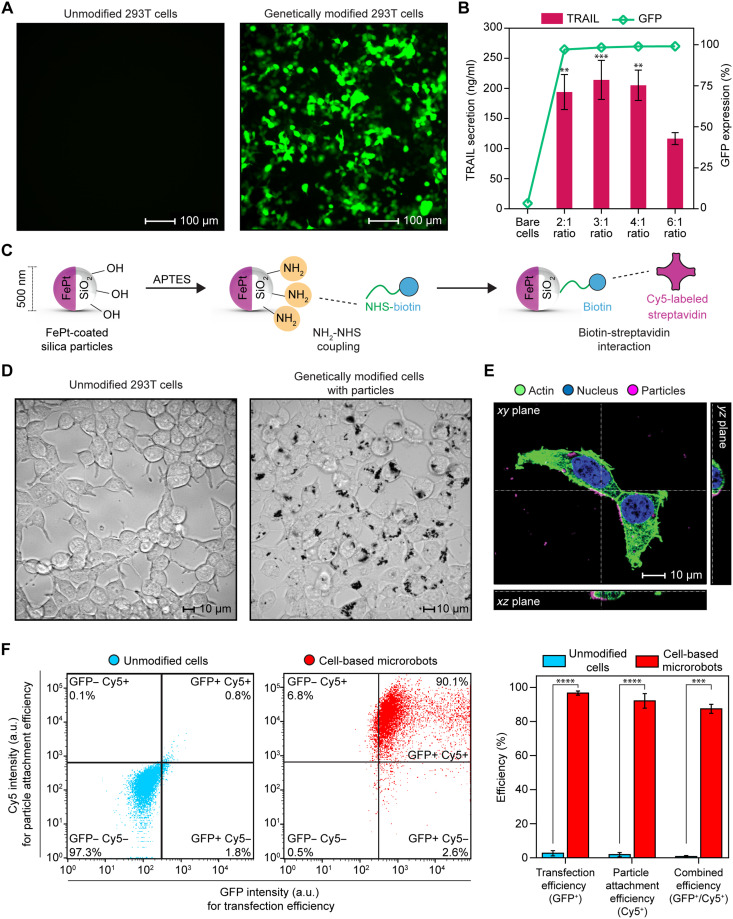
Optimization and characterization of cell-based microrobots. (**A**) The transfection efficiency of 293T cells was optimized by varying the reagent-to-DNA ratio (2:1, 3:1, 4:1, and 6:1). Representative fluorescence images were acquired using the optimized 2:1 ratio; images from all conditions, including the 2:1 ratio shown here, are provided in fig. S8. Scale bars, 100 μm. (**B**) Flow cytometry showed consistent GFP expression across all ratios. Data are presented as the means ± SD, *n* = 3 technical replicates. ELISA analysis revealed comparable TRAIL secretion at 2:1, 3:1, and 4:1 ratios, with a significant decrease at a 6:1 ratio. Data are presented as the means ± SD from *n* = 4 technical replicates. One-way ANOVA with Tukey’s post hoc test: **P < 0.01 and ***P < 0.001. Significant differences were found for 2:1 versus 6:1 (**), 3:1 versus 6:1 (***), and 4:1 versus 6:1 (**); all other comparisons between ratios were not significant. (**C**) Schematic illustrating the functionalization of magnetic Janus particles through sequential chemical reactions. (**D**) Phase-contrast microscopy images confirming successful conjugation of magnetic Janus particles to 293T cells. For a better comparison of each modification step, the corresponding images are also provided in fig. S7. Scale bars, 10 μm. (**E**) Confocal Z-stack imaging of cell-based microrobots revealed the accumulation of magnetic Janus particles at the outer membrane of 293T cells. Scale bar, 10 μm. (**F**) Flow cytometry analysis shows a transfection efficiency (GFP^+^) of 96.6 ± 1.2% and a particle attachment efficiency (Cy5^+^) of 92.1 ± 4.3%. The overall yield of fully functional microrobots (GFP^+^/Cy5^+^) reached 90.1% in a representative replicate, with an average of 87.4 ± 2.7% of all replicates. (a.u., arbitrary units; *n* = 10,000 events per group for flow cytometry). Data are represented as the means ± SD from *n* = 3 independent biological replicates. Student’s *t* test: ***P < 0.001 and ****P < 0.0001.

In the first step, different transfection reagent-to-DNA ratios were tested for GFP expression: 2:1, 3:1, 4:1, and 6:1 ratios. The expression of GFP was assessed by fluorescence microscopy and flow cytometry 48 hours after transfection. GFP expression was detectable in all ratios, with no visual differences among the groups (Fig. 2A and fig. S8). As shown by flow cytometry analysis, the 6:1 ratio produced slightly higher GFP expression than the others, with expression levels above 97% at 48 hours posttransfection across all groups (Fig. 2B). In addition to optimizing the transfection reagent-to-DNA ratio, we further investigated the most effective transfection duration. We measured GFP expression after transfection at 24, 48, and 72 hours using a 2:1 reagent-to-DNA ratio. A time-dependent increase in GFP expression was observed using fluorescence microscopy and flow cytometry analyses, indicating that transgene expression gradually increased over time; however, no statistically significant differences were observed between time points (fig. S9).

Although optimizing GFP expression provided valuable insight into transfection efficiency, the primary goal of this study was to maximize TRAIL expression to induce a high degree of cancer cell death. To achieve this, TRAIL expression levels were measured by enzyme-linked immunosorbent assay (ELISA) at 24, 48, and 72 hours posttransfection. We measured TRAIL levels as 85 ng/ml at 24 hours, 211 ng/ml at 48 hours, and 188 ng/ml at 72 hours. Given that the highest level of TRAIL expression was observed at 48 hours, all subsequent experiments were conducted at this time point (fig. S10). As a next step, TRAIL levels were compared across different transfection reagent-to-DNA ratios (2:1, 3:1, 4:1, and 6:1) at 48 hours posttransfection. The results showed no significant difference between 2:1, 3:1, and 4:1 ratios. The 6:1 group, however, showed a substantial reduction in TRAIL secretion. On the basis of these results, the 2:1 ratio at 48 hours posttransfection was selected for all subsequent experiments to reduce reagent consumption, increase transfection efficiency, and increase gene expression (Fig. 2B).

Next, transfected cells were conjugated with magnetic Janus particles to enable magnetic control of cell swarms. First, we functionalized FePt-coated silica particles to characterize the successful attachment of particles. After sputtering a 60-nm-thick FePt layer, silica particles were functionalized with amino (─NH_2_) groups, then conjugated with NHS (*N*-hydroxysuccinimide)-biotin, and subsequently bound to Cy5-labeled streptavidin through strong biotin-streptavidin interactions (Fig. 2C). As a result of this modification, magnetic Janus particles became fluorescently labeled and detectable in the Cy5 fluorescence channel. After staining, magnetic Janus particles were added to the genetically modified 293T cell culture medium. Phase-contrast microscopy images confirmed successful binding of the magnetic Janus particles to 293T cells, with an affinity for attachment to the cell membrane rather than freely accumulating on the cell culture plate (Fig. 2D and fig. S7). Because of their nanoscale size, the particles are capable of being internalized by cells; therefore, we investigated whether they were taken up by 293T cells. In contrast to our previous experiments on macrophages, which internalized Janus particles through phagocytosis (*12*, *15*), confocal Z-stack imaging showed that 293T cells retained the particles on their outer membranes (Fig. 2E).

The resultant genetically modified and particle-attached cell–based microrobots were examined at multiple time points (24, 48, 72, and 96 hours posttransfection) to identify the optimal therapeutic windows in terms of TRAIL secretion, GFP expression, and magnetic particle retention. As a result, 48- to 96-hour posttransfection appeared to correspond to a therapeutic window that exhibited peak TRAIL secretion (>200 ng/ml), high particle retention (>85%), and a high degree of transfection efficiency (>85%) (fig. S11). According to these results, 48-hour posttransfection was selected for subsequent experiments, given that 24 hours produced insufficient TRAIL, while later time points did not show significant improvements in TRAIL secretion, particle retention, or transfection efficiency. Therefore, we determined that 48 hours would be the most effective time for testing.

On the basis of the collected data, the optimized fabrication process and timeline of the cell-based microrobots are schematically illustrated (fig. S12). The culture media from genetically modified 293T cells were collected at 24 hours posttransfection. Magnetic Janus particles were added to this medium at a concentration of 0.2 mg/ml and incubated further with 293T cells overnight. Cell-based microrobots were considered successfully formed at 48 hours posttransfection as of experimental day 5 (fig. S12). To evaluate the production efficiency of the cell-based microrobots, flow cytometry was performed as the final characterization step. Flow cytometry analysis shows an average transfection efficiency (GFP^+^ cells) of 96.6 ± 1.2% and a particle attachment efficiency (Cy5^+^ cells) of 92.1 ± 4.3%. The overall yield of fully functional microrobots—defined as cells positive for both GFP (indicating successful transfection) and Cy5 (indicating successful particle attachment) (GFP^+^/Cy5^+^ cells)—was 90.1% in the representative biological replicate, with an average yield of 87.4 ± 2.7% across three independent biological experiments (Fig. 2F). Although 48-hour posttransfection was selected for subsequent experiments, we also evaluated long-term TRAIL secretion of the cell-based microrobots. The results showed a high level of TRAIL secretion observed during the early posttransfection period in serum-containing culture medium, peaking at experimental day 5, followed by a gradual decline in daily production and reaching a plateau from day 8 onward (fig. S13).

Last, the in vitro safety of the cell-based microrobots was investigated using macrophage coculture experiments. The results indicate that direct exposure to cell-based microrobots did not induce macrophage activation (fig. S14); however, further in vivo studies are required to evaluate their biosafety fully.

### Microrobots selectively kill cancer cells while preserving healthy cells

We first evaluated the selective cancer-killing ability of the cell-based microrobots using a conditioned-medium approach. Conditioned medium collected 48 hours posttransfection contained TRAIL at an average of 220 ng/ml, whereas medium from unmodified 293T cells contained no detectable TRAIL. These conditioned media were subsequently applied to both healthy and cancer cell lines to evaluate cancer-selective cytotoxicity (figs. S15 and S16). BJ human fibroblasts and hAMSCs were used as healthy cell lines, while cancerous cells were HCT116 colon cancer, T98G glioblastoma, ACHN renal carcinoma, and ONCO-DG-1 ovarian carcinoma.

In healthy cells, exposure to the conditioned medium had no reduction in viability, confirming the negligible cytotoxicity of TRAIL and other factors secreted by cell-based microrobots (fig. S15). However, cancer cell viability was significantly reduced across all tested cancer cell lines, with viability dropping to 8% in HCT116 colon cancer cells, 2% in T98G brain cancer cells, 10% in ACHN renal carcinoma cells, and 48% in ONCO-DG-1 ovarian carcinoma cells (fig. S16). These findings highlight the ability of cell-based microrobots to selectively eliminate diverse cancer cell types without harming healthy cells. This varying sensitivity of the cancer cell lines is consistent with known differences in responsiveness to TRAIL-induced apoptosis. To further investigate this variability, we analyzed IC_50_ dose-response curves for commercially available recombinant human TRAIL in both 2D and 3D cultures, where IC_50_ values represent the TRAIL concentration required for 50% loss of cell viability. These data provide an additional reference for understanding the intrinsic differences in TRAIL susceptibility among the tested cancer cell lines (figs. S17 and S18).

An important consideration for translating this platform toward therapeutic applications is the heterogeneous sensitivity of tumors to TRAIL, as some cancer types can exhibit partial or complete resistance (*43*). Our microrobots can address the challenge of partially resistant cells by providing localized TRAIL release at elevated concentrations directly around the tumor site, where prolonged exposure can sensitize semiresistant cancer cells (e.g., ONCO-DG-1) and yield meaningful antitumor effects (fig. S19). However, tumors that are fully resistant to TRAIL may require future implementation of this microrobot platform in combination with sensitizing agents or pathway-targeted therapeutics to effectively overcome TRAIL resistance.

**Fig. 3.**
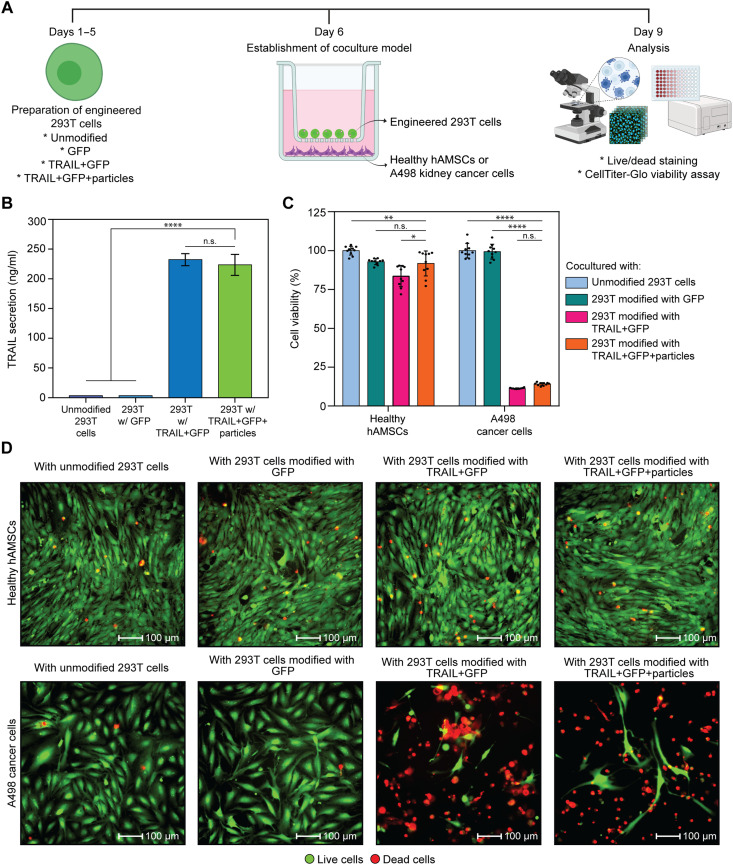
Cell-based microrobots selectively kill cancer cells without damaging healthy cells. (**A**) Schematic illustration of the experimental setup with four groups: (i) unmodified 293T cells, (ii) 293T cells modified with *GFP* only, (iii) 293T cells modified with *TRAIL* + *GFP*, and (iv) cell-based microrobots—293T cells modified with *TRAIL* + *GFP* and then conjugated to magnetic Janus particles. 293T cells were seeded in the upper chamber of a transwell system; healthy hAMSCs or A498 cancer cells were seeded in the lower chamber. Cell viability was assessed after 3 days of coculture. Created in BioRender. N. O. Dogan (2026), https://biorender.com/3lke7fg. (**B**) TRAIL secretion was measured in all groups at 48 hours posttransfection. Data are presented as the means ± SD from *n* = 3 technical replicates. One-way ANOVA with Tukey’s post hoc test; n.s. indicates *P* ≥ 0.05, and ****P < 0.0001. (**C**) CellTiter-Glo viability assays showing a minimal effect on healthy hAMSC viability across groups, while A498 cancer cells exhibited significant death in both TRAIL + GFP and TRAIL + GFP + particles groups. Data are presented as the means ± SD from *n* = 10 technical replicates. One-way ANOVA with Tukey’s post hoc test; n.s. indicates *P* ≥ 0.05; *P < 0.05, **P < 0.01, and ****P < 0.0001. (**D**) Representative live/dead fluorescence images confirming the selective cytotoxicity of cell-based microrobots on A498 cancer cells, with no detectable harm on healthy hAMSCs. Scale bars, 100 μm.

Next, we investigated the selective cancer cell–killing capability of the microrobots using a coculture system that enables direct cell-to-cell communication. To achieve this, we used transwell inserts with 0.4-μm pore sizes, commonly used in drug transport studies, to physically separate cell populations while allowing soluble factors to diffuse through (*60*). Four distinct 293T cell lines were established to specifically test whether selective cancer cell death was caused by TRAIL secretion instead of genetic manipulation or particle conjugation: (i) unmodified 293T cells, (ii) cells transfected with a plasmid encoding only *GFP* (*GFP*), (iii) cells transfected with a plasmid encoding both *TRAIL* and *GFP* (*TRAIL* + *GFP*), and (iv) cell-based microrobots—cells transfected with a plasmid encoding both *TRAIL* and *GFP* and subsequently conjugated with magnetic Janus particles (*TRAIL* + *GFP* + particles). A five-day process was used to prepare these experimental groups (Fig. 3A and fig. S12). First, quantitative polymerase chain reaction analysis revealed that 293T cells transfected with *TRAIL* + *GFP* showed an ~239-fold increase in *TRAIL* mRNA expression compared with *GFP*-transfected control cells (fig. S20). Next, conditioned media were harvested at 48 hours posttransfection to quantify TRAIL secretion. As expected, TRAIL was undetectable in the media collected from both unmodified and *GFP*-modified 293T cells, confirming that TRAIL was explicitly produced in response to intended genetic modification and not from unintended expression or nonspecific transfection effects. TRAIL expression levels were significantly elevated in the TRAIL group and remained unaffected by the presence of magnetic Janus particles, as similar concentrations were observed in both TRAIL + GFP and TRAIL + GFP + particles groups (Fig. 3B).

Following that, the engineered 293T cells were detached, counted, and seeded into the upper chambers of the transwell system, while the lower chambers were seeded with either healthy hAMSCs or A498 renal carcinoma cells (Fig. 3A). Viability analysis demonstrated that healthy hAMSCs were largely unaffected, with viability decreasing to 91% after coculturing with 293T cells modified with TRAIL + GFP + particles. In contrast, A498 cancer cells exhibited pronounced cell death, as their viability dropped to 14% after coculturing with 293T cells modified with TRAIL + GFP + particles (Fig. 3C). Live/dead fluorescence images of healthy hAMSCs and A498 cancer cells further confirmed the selective cytotoxicity of TRAIL-secreting cell–based microrobots, demonstrating selective killing of cancer cells with no detectable damage to healthy cells (Fig. 3D).

### Magnetic actuation and steering of cell-based microrobots

For magnetic control, we used an electromagnetic setup to generate uniform, rotating magnetic fields with a strength ranging from 1 to 20 mT within the workspace. These rotating magnetic fields create magnetic torque on cell-based microrobots, therefore enabling their controlled locomotion. Given that fully magnetically coated particles are symmetrical, they tend to align with the field without producing sufficient torque, thus producing no directional motion. To overcome this, we used Janus particles—microspheres with only one hemisphere coated with magnetic FePt material. As a result of this asymmetric design, structural anisotropies are introduced, which are important for torque generation (*61*). Because of its magnetic anisotropy, the FePt-coated side aligns its magnetic easy axis with the external field. Janus particles attached to the cell surface generate torque, which is transferred to the entire cell, causing it to roll on the surface. In addition, we were able to precisely control the rolling direction of the cell-based microrobots by adjusting the orientation of the magnetic field.

**Fig. 4.**
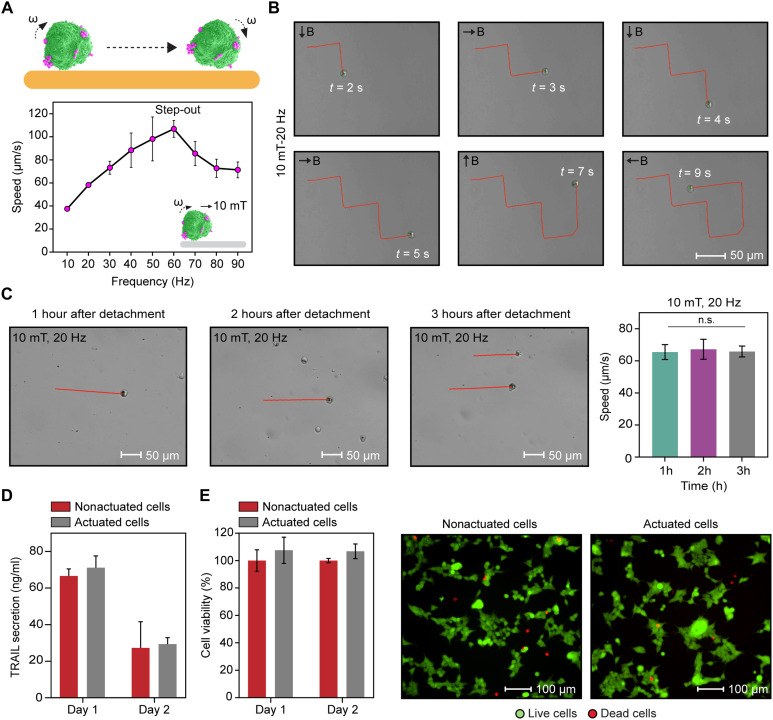
Magnetic actuation of cell-based microrobots and postactuation characterizations. (**A**) Cell-based microrobots were magnetically controlled along predefined trajectories within a microchannel, reaching a step-out frequency of 60 Hz with an average speed of 107 μm/s under a 10-mT rotating magnetic field. Data are presented as the means ± SD, with at least *n* = 3 technical replicates. (**B**) Magnetic control of cell-based microrobots was achieved by changing the direction of the applied magnetic field (10 mT) at a frequency of 20 Hz. Vector B represents the magnetic field direction. Scale bar, 50 μm. (**C**) Long-term motility analysis of microrobots up to 3 hours (h) after detachment. Microrobots maintained stable velocity without noticeable particle detachment or loss of magnetic responsiveness. Data are presented as the means ± SD from *n* = 3 technical replicates. One-way ANOVA with Tukey’s post hoc test; n.s. indicates *P* ≥ 0.05 for all comparisons. Scale bars, 50 μm. (**D**) TRAIL secretion of microrobots after repeated magnetic actuation, where the actuation was performed under 10-mT, 20-Hz rotating magnetic fields for 5 min, repeated for 2 days. Data are presented as the means ± SD from *n* = 4 technical replicates. (**E**) 293T cell viability of microrobots after repeated magnetic actuation (actuation performed under 10-mT, 20-Hz rotating magnetic fields for 5 min, repeated for 2 days). CellTiter-Glo assay, which showed no cell death following magnetic actuation, indicating that the cells tolerate magnetic control and surface-rolling locomotion well. Data are presented as the means ± SD, with at least *n* = 7 technical replicates. Viability was also assessed using live/dead staining, which showed that most microrobots remained viable. Scale bars, 100 μm.

Despite the magnetic Janus particles only being attached to the surface of the 293T cells, effective magnetic actuation and control were achieved without compromising cellular function. We first analyzed the translational speed of cell-based microrobots and determined their step-out frequency. Below a certain threshold, the translational speed of microrobots increases approximately linearly with the applied magnetic field frequency. As soon as this resistance threshold is exceeded, the microrobots are no longer able to maintain synchronous motion with the external magnetic field because of increasing fluid resistance. As a result, the velocity drops sharply at a threshold known as the step-out frequency (*62*). To quantify this threshold, we gradually increased the rotational frequency of a 10-mT magnetic field while measuring the corresponding translational velocity of microrobots. A maximum average speed of 107 μm/s was achieved at 60 Hz, which was identified as the step-out frequency (Fig. 4A and movie S1). Although magnetic Janus particle attachment to engineered cells was performed without external magnetic prealignment, resulting in heterogeneous particle orientations, we still obtained relatively high velocity distributions, as shown in Fig. 4A. Despite this heterogeneity, the cell-based microrobots responded robustly to rotating magnetic fields and achieved high propulsion speeds, indicating that effective magnetic actuation can be obtained even without uniform particle orientation. However, for future in vivo studies, an external magnetic field could be applied during the particle-attachment step to prealign the magnetic Janus particles, which may further reduce velocity variability and enable more standardized motion in complex biological environments.

As a next step, we demonstrated that an individual cell-based microrobot could be rolled and steered along predefined trajectories in multiple directions within microchannels under an applied magnetic field of 10 mT at 20 Hz (Fig. 4B and movie S2). We then investigated swarms of cell-based microrobots under rotating magnetic fields and successfully guided multiple cells to target directions (movie S3). Although transient clustering may occur under static, confined microchannel conditions because of high microrobot concentration and prolonged residence time, it can be mitigated by dilution, reduced residence time, and antifouling surface modification to particles, enabling well-dispersed swarm movements relevant to in vivo applications. Last, we also assessed microrobot motility over extended periods, up to 3 hours after detachment. Throughout this duration, the microrobots maintained stable movement with no particle detachment, no reduction in velocity, and no loss of magnetic responsiveness (Fig. 4C and movie S4).

Once the cell-based microrobots reach the target tissue, they must remain viable to perform their biological functions, which involve secreting TRAIL to selectively induce apoptosis in cancer cells. We therefore assessed cell viability and functional activity following magnetic control. Cell-based microrobots were steered for 5 min at 20 Hz under a 10-mT rotating magnetic field, with repeated actuation performed over two consecutive days. After each daily actuation cycle, cells were collected and incubated overnight at 37°C in a humidified atmosphere with 5% CO_2_. The TRAIL secretion was measured after overnight incubation and compared with that of nonactuated controls, given that protein synthesis can be transiently suppressed by stress associated with repeated passaging and trypsinization in transiently transfected cells. TRAIL levels measured after each actuation cycle were similar to those of nonactuated but passaged cells, indicating that magnetic actuation does not adversely affect TRAIL secretion (Fig. 4D). Consistently, viability assays showed no reduction in cell viability following repeated magnetic actuation (Fig. 4E). These results demonstrate that cell-based microrobots remain functionally alive and capable of secreting TRAIL after multiple days of repeated magnetic control. Future studies could use viral transduction strategies instead of nonviral transient transfection to achieve more stable gene expression and to further minimize potential variability in protein secretion associated with cell passaging.

### Targeting and inducing apoptosis in tumor spheroids

**Fig. 5.**
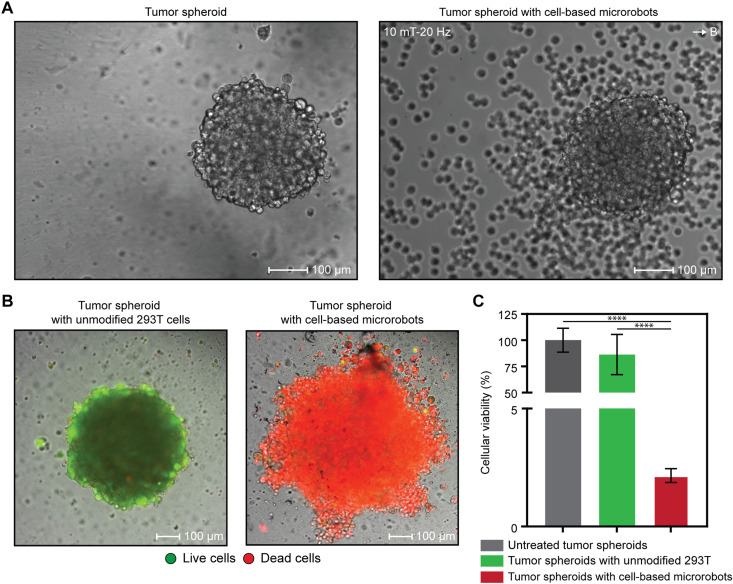
Cell-based microrobots target and selectively kill 3D tumor spheroids in vitro. (**A**) Differential interference contrast imaging demonstrating the magnetic guidance of cell-based microrobots toward a 3D tumor spheroid. Scale bars, 100 μm. (**B**) As shown by live/dead fluorescence imaging, conditioned medium from cell-based microrobots induced increased tumor cell death in 3D A498 renal adenocarcinoma tumor spheroids, unlike medium from unmodified 293T cells. Scale bars, 100 μm. (**C**) CellTiter-Glo 3D assay revealed significantly reduced tumor spheroid viability after 24 hours of incubation with conditioned medium from cell-based microrobots compared to medium from unmodified 293T cells. Data are presented as the means ± SD, *n* = 6 technical replicates. One-way ANOVA with Tukey’s post hoc test; ****P < 0.0001.

In contrast to conventional 2D monolayer cultures, 3D tumor spheroids more accurately replicate the in vivo tumor microenvironment, including key features such as cell-cell interactions, nutrient gradients, and drug diffusion barriers (*63*). Therefore, assessing microrobot performance in this model is more clinically relevant to understanding their therapeutic potential. For this analysis, 3D tumor spheroids were generated by culturing A498 human renal adenocarcinomas in ultralow-attachment plates. The low adhesion surface of the cultureware inhibited cell attachment to the plate, promoting cell-to-cell aggregation through naturally secreted extracellular matrix components and resulting in the formation of 3D tumor spheroids. To investigate the therapeutic capabilities of cell-based microrobots, we used magnetic guidance to direct microrobot swarms toward the 3D tumor spheroids (movie S5). The results confirmed successful navigation and targeted accumulation of microrobots around the spheroid structures (Fig. 5A). The antitumor efficacy of the treatment was also evaluated. It was found that the unmodified 293T cells exhibited no significant cytotoxicity, whereas the cell-based microrobots efficiently induced 3D tumor cell death (Fig. 5, B and C). As a result of these findings, our microrobot system offers the potential for targeted delivery and localized therapy in physiologically relevant 3D tumor models.

Although 293T cells were used in this study as the in vitro cell source, the microrobot platform is inherently adaptable and can be extended to clinically relevant cell types. To demonstrate this flexibility and improve translational relevance, we also engineered human skin fibroblasts as an alternative therapeutic cell source. Fibroblasts were selected because they are clinically meaningful and safe cell types that are abundant in the human body, can be readily isolated from minimally invasive skin biopsies, and naturally migrate toward sites of tissue damage and inflammation as part of the wound-healing process (*64*, *65*). These human fibroblasts were efficiently engineered using multiple viral vectors to secrete TRAIL at levels sufficient to induce cancer cell death (fig. S21). Furthermore, we further incorporated a doxycycline-inducible expression system to enable controlled, on-demand TRAIL secretion to further reduce the risk of unintended cytotoxicity (fig. S22). Given that doxycycline-mediated induction requires sustained cellular exposure (~48 hours), one potential in vivo strategy would be to preload the cell-based microrobots with doxycycline-loaded magnetic particles rather than relying on systemic administration. By carrying these particles, cells would be exposed to continuous doxycycline from the time of preparation through their migration and accumulation at the tumor site. Nevertheless, the efficiency of this strategy will depend on optimized doxycycline loading and controlled release kinetics, which require systematic investigation. Moreover, spatial specificity in vivo would still depend on effective microrobot localization and retention at the target site, which needs to be systematically investigated in future in vivo studies.

## DISCUSSION

In this study, we developed a living engineered human cell–based biohybrid microrobotic system. Robotic mobility is integrated with genetic engineering to selectively kill multiple cancer cell types while causing no damage to healthy cells. This selectivity arises from the mechanism by which TRAIL binds to the special cell receptors (DR4 and DR5), initiating a signaling pathway that ultimately leads to cell death. A key advantage is that cancer cells express these receptors at high levels and are therefore highly sensitive to TRAIL-induced cell death, while healthy cells express them at much lower levels and remain largely resistant even at elevated TRAIL concentrations. This selective cancer cell–killing ability without harming healthy cells is clearly demonstrated in our experimental results (Fig. 3, C and D, and figs. S15 and S16).

Next, magnetic actuation was integrated into our system to guide and concentrate the TRAIL-secreting cells near the tumor region, thereby increasing the local density of therapeutic cells. Through this targeted positioning, TRAIL can be released closer to tumor cells, rather than circulating systemically throughout the body. Given that TRAIL is a diffusible cytokine, it can still induce apoptosis in nearby cancer cells even if the cell-based microrobots do not remain anchored within the tumor. Here, for magnetic control, we used FePt-coated magnetic Janus particles, which are known to be adsorbed by the reticuloendothelial system in the liver and spleen, where they undergo gradual degradation or are stored there until they have been completely broken down over time, depending on their coating, size, and surface properties (*50*, *66*).

We initially validated our platform using human 293T cells, a robust and reproducible in vitro model ideal for testing FePt particle attachment, genetic engineering, and functional performance. Using these cells, we established magnetically steerable biohybrid microrobots capable of secreting TRAIL for several days. To improve translational relevance, we next engineered human skin fibroblasts as an alternative cell type. These human fibroblasts were efficiently modified using multiple viral vectors to produce TRAIL at levels sufficient to induce cancer cell death (figs. S21 and S22), demonstrating the adaptability of the platform across different cell types. For most cell-based therapies, particularly those intended as delivery vehicles for therapeutic agents, long-term engraftment is not required and often not observed. Instead, injected cells typically exhibit transient persistence, followed by immune-mediated clearance, with therapeutic efficacy arising from short-term functional activity rather than permanent integration. Thus, localized delivery of elevated TRAIL amounts can achieve therapeutic benefit without requiring permanent cell integration. For future in vivo applications, additional therapeutic cell sources such as mesenchymal stem cells or patient-derived autologous cells may also be considered and explored, with careful consideration of the long-term fate of cells, immune interactions, and clearance profiles appropriate for the intended therapeutic application.

Despite the success demonstrated in our in vitro studies, further investigations are needed to translate these capabilities into therapeutic applications and fully realize the potential of TRAIL-secreting human cell–based microrobots. Future in vivo studies should explore microrobot delivery through clinically relevant routes and evaluate their behavior in physiological environments. Initial feasibility testing may be performed via intratumoral administration, enabling direct evaluation of safety, tissue retention, and local therapeutic efficacy. This may be followed by arterial delivery to tumor-feeding vessels to assess the magnetic navigation and targeting capabilities of microrobots under physiological conditions. Upon successful demonstration of robust magnetic control and targeting efficiency, systemic intravenous administration may also be investigated. In addition, although in vitro coculture experiments with macrophages did not induce macrophage activation upon direct exposure to cell-based microrobots (fig. S14), further in vivo investigations are required to evaluate their biosafety fully. Such future in vivo studies should assess long-term biocompatibility and safety, including biodistribution, potential uncontrolled cell proliferation, and interactions with the host immune system. Furthermore, the fate of the magnetic Janus particles must also be investigated, including possible accumulation in off-target organs and long-term clearance. Additional studies will be required to assess the impact of extracellular matrix barriers, elevated interstitial fluid pressure, and TRAIL stability in vivo, as well as to evaluate retention, detachment under physiological shear stresses, and penetration within tissue-mimicking matrices. Last, scaling external magnetic fields for in vivo navigation presents additional translational challenges. Future studies must address field strength, spatial control, and the risk of tissue heating or unintended effects in tissues. Addressing these factors will be essential for translating this microrobotic platform into a safe and effective therapeutic modality.

The strategy presented here is highly flexible, allowing both the therapeutic cell type and therapeutic gene to be easily substituted, which facilitates adaptability to different disease models. Because of its versatility, the platform can be adapted to a wide range of biomedical applications while maintaining a high degree of specificity and targeting. In summary, this study represents a substantial advance in microrobotics by integrating physical targeting with biological selectivity. We demonstrate that magnetically guided, cell-based microrobots enable localized, high-level delivery of therapeutic agents, which selectively eliminate multiple cancer cell types while preserving healthy cells.

## MATERIALS AND METHODS

### Fabrication of magnetic Janus particles

The magnetic Janus particles were fabricated from 500-nm-diameter silica beads (microParticles GmbH), which were then partially coated with a 60-nm-thick FePt magnetic nanofilm. Silica beads were first dispersed on oxygen plasma–treated silicon wafers. Molecular beam epitaxy was used to deposit a 60-nm-thick FePt layer onto one hemisphere of the particles under ultrahigh vacuum conditions (~2 × 10^−9^ to 5 × 10^−9^ mbar), followed by annealing at 680°C for an hour. Previous reports have provided detailed structural and compositional characterization of magnetic Janus particles (*12*, *15*). Afterward, the Janus particles were ultrasonically detached from the silicon wafer and collected for further surface functionalization.

### Fluorescent labeling of magnetic Janus particles

A sequential surface modification protocol was used to label magnetic Janus particles fluorescently (Fig. 2C). As a first step, APTES (3-aminopropyltriethoxysilane; Sigma-Aldrich) was used to introduce amine groups (─NH_2_) to the silica surfaces. For this, the particles were vortexed in 0.05% (v/v) APTES in ethanol for 3 hours and then incubated at 65°C for an hour. After several washes with ethanol and dimethyl sulfoxide (DMSO; Sigma-Aldrich), the amine-functionalized particles were biotinylated for 3 hours in DMSO with EZ-Link NHS-biotin (5 mg/ml; Thermo Fisher Scientific). Subsequent washing with DMSO and Dulbecco’s phosphate-buffered saline (DPBS; Gibco) was applied to remove excess reagents. Last, biotinylated surfaces were labeled with the Cy5 dye by incubating the particles in a 50 μg/ml solution of Cy5-labeled streptavidin (Alexa Fluor 647 conjugate, Thermo Fisher Scientific) in DPBS for 1 hour with vortexing. A series of DPBS washes was used to remove unbound dye. Following the preparation of fluorescent magnetic Janus particles, they were sterilized by ultraviolet light and stored for further use.

### Characterization of magnetic Janus particles

SEM analysis was performed on FePt-coated silica Janus particles dispersed on clean glass substrates coated with 10-nm gold layers using a benchtop sputter coater (Leica EM ACE600, Leica Microsystems) to enhance surface conductivity and imaging contrast. A Zeiss Ultra 550 Gemini microscope (Carl Zeiss Inc.) equipped with an Everhart-Thornley secondary electron (SE2) detector was used to perform SEM imaging and EDX analysis.

### Cell culture

293T human embryonic kidney cells (DSMZ), BJ human fibroblasts [American Type Culture Collection (ATCC)], hAMSCs (Cellular Engineering Technologies), HCT116 human colorectal carcinoma cells (ATCC), and T98G human glioblastoma cells (ATCC) were maintained in high-glucose Dulbecco’s modified Eagle’s medium (Gibco). ACHN and A498 human renal adenocarcinomas (ATCC) and ONCO-DG-1 human ovarian adenocarcinomas (DSMZ) were cultivated in Roswell Park Memorial Institute (RPMI) 1640 medium (Gibco). All culture media were supplemented with 10% fetal bovine serum (Gibco) and 1% penicillin-streptomycin (Gibco). Cells were cultured in sterile 75-cm^2^ polystyrene cell culture flasks at 37°C in a humidified incubator with 5% CO_2_. Passaging was performed with 0.25% (w/v) trypsin-EDTA solution once the cells reached 80% confluency.

### Preparation of plasmid DNAs

Chemically competent *Escherichia coli* One Shot Stbl3 cells (Thermo Fisher Scientific) were transformed with the plasmids to amplify plasmid DNA, which were previously described (*39*, *67*). Ampicillin was added to the culture medium at a final concentration of 100 μg/ml for selection. Plasmid DNA was extracted using the NucleoBond Xtra Plasmid Purification Kit (Macherey-Nagel).

### Optimization of transfection efficiency

To optimize transfection efficiency, 293T cells were seeded on day 1 into six-well plates at a density of 3 × 10^5^ cells per well in 2 ml of standard culture medium. On day 2, cells were transfected with a complex of plasmid DNA and FuGENE 6 transfection reagent (Promega), prepared according to the manufacturer’s protocol. The optimal transfection conditions were determined by testing a range of transfection reagent-to-DNA ratios (2:1, 3:1, 4:1, and 6:1; table S3). Transfection complexes were directly added to the existing culture medium without medium replacement. After overnight incubation, the transfection medium was replaced with fresh culture medium to maintain cell viability and support protein expression. Cells were then kept in culture until day 5 to allow sufficient time for transgene expression. On day 5, transfected cells were initially imaged using a fluorescence microscope (Nikon Eclipse Ti-E) under the fluorescein isothiocyanate channel [excitation/emission (Ex/Em): 488/520 nm] to visualize GFP expression. As a next step, conditioned media from transfected cells were collected and analyzed by ELISA. Human CD253 (TRAIL) ELISA kits (BD Biosciences) were used to measure the levels of secreted TRAIL, following the manufacturer’s instructions. Absorbance measurements were made using a microplate reader (Tecan Infinite M Plex). A representative human CD253/TRAIL ELISA standard curve is shown in fig. S23, and the concentrations of samples were calculated on the basis of the fitted calibration equation to determine the amount of secreted TRAIL.

Parallel to this, flow cytometry (BD LSRFortessa X-20, BD Biosciences) was used to quantify GFP^+^ cells. The adherent cells on culture plates were washed, detached with trypsin-EDTA, and then fixed with 4% paraformaldehyde (PFA; v/v in DPBS) for 20 min. The cells were subsequently analyzed using a blue laser (Ex/Em: 488/525 nm) in a flow cytometer. In each group, 10,000 events were collected and analyzed using FlowJo software (version 10.8.1). A representative flow cytometry gating strategy is shown in fig. S24, in which cells were first gated based on FSC-A (forward scatter area) and SSC-A (side scatter area), followed by singlet selection, and subsequently gated for GFP^+^ and Cy5^+^ populations. On the basis the optimization results, a 2:1 reagent-to-DNA ratio at 48 hours posttransfection was selected for all subsequent experiments.

### Preparation and characterization of cell-based microrobots

The cell-based microrobots were developed using the same protocol described in the previous section, with one modification introduced on day 4. In particular, on day 4, conditioned media from transfected 293T cells (24 hours of posttransfection) were collected and supplemented with magnetic Janus particles at a concentration of 0.2 mg/ml. To facilitate effective surface attachment, nanoparticle-enriched media were incubated with 293T cells for 24 hours. As of day 5 (fig. S12), fully assembled cell-based microrobots had been generated and visualized using phase-contrast microscopy imaging (Leica SP8 confocal microscope, Leica Microsystems). These microrobots were subsequently used for further characterization.

To assess 293T cell viability after each modification step, four experimental groups were prepared: (i) unmodified cells, (ii) genetically modified cells without particles, (iii) unmodified cells with particles, and (iv) genetically modified cells with particles. These groups were established following the previously described protocol, with minor modifications based on the treatment conditions. For genetically modified cells without particles, no particles were added on day 4. For unmodified cells with particles, no plasmid DNA was introduced; instead, on day 4, the cell medium was replaced with particle-containing medium, and incubation continued until day 5. The CellTiter-Glo Luminescent Cell Viability Assay (Promega) was performed for all groups on day 5, according to the manufacturer’s instructions. Luminescence values were measured in a 96-well black/clear-bottom plate (Thermo Fisher Scientific) using a microplate reader (Tecan, Infinite M Plex).

SEM imaging was performed using cell-based microrobots fixed with 4% PFA for 20 min in DPBS, followed by dehydration for 10 min with increasing ethanol concentrations (30, 50, 75, 90, and 100%). After that, the cells were chemically dehydrated for 10 min with increasing concentrations of hexamethyldisilazane (Sigma-Aldrich) solutions diluted in ethanol (33, 50, 67, and 100% hexamethyldisilazane). A fume hood was used to dry the samples overnight under air pressure. A benchtop sputter coater (Leica EM ACE600, Leica Microsystems) was used to sputter 10-nm gold over the air-dried samples. SEM imaging was conducted using a Zeiss Ultra 550 Gemini SEM (Carl Zeiss Inc.) at an accelerating voltage of 3 keV with an SE2 detector. The pseudocolored images were created using Adobe Photoshop (version 21.0).

For confocal imaging, cell-based microrobots were chemically fixed in 4% (v/v) PFA (Alfa Aesar) in DPBS for 20 min at ambient temperature. Afterward, 0.1% (v/v) Tween 20 (Sigma-Aldrich) in DPBS was used to permeabilize membranes for 30 min to facilitate intracellular staining. Cells were stained with a solution containing Hoechst 33342 (1 μg/ml; Thermo Fisher Scientific) for nucleus staining and ActinRed 555 ReadyProbes (2 drops/ml; Life Technologies) for actin filament staining for 30 min in the dark to prevent photobleaching. A Leica SP8 confocal laser scanning microscope was used to capture high-resolution fluorescence images, enabling visualization of nuclear and cytoskeletal structures, as well as magnetic Janus particles.

A quantitative analysis of cell-based microrobot populations was conducted using flow cytometry (BD LSRFortessa X-20, BD Biosciences). A comparison was performed between bare 293T cells and cell-based microrobots. All groups were detached from the surface and fixed with PFA [4% (v/v) in DPBS] for 20 min. After rinsing with DPBS, cells were characterized using a blue laser (Ex/Em: 488/525 nm) to detect the GFP-expressing transfected 293T cells, and a red laser (640/730 nm) to detect magnetic Janus particles. FlowJo 10.8.1 was used to analyze the data, with 10,000 events collected per group.

### Selective induction of apoptosis in human cancer cell lines through coculture experiments

For coculture experiments, polystyrene plates (Corning) were used with transwell inserts (6.5-mm-diameter, 0.4-μm-pore-size polycarbonate membrane; tissue culture treated and sterile). The upper chamber was seeded with (i) unmodified 293T cells, (ii) 293T cells engineered to express *GFP* only, (iii) 293T cells engineered to express *TRAIL* + *GFP*, and (iv) 293T cells engineered to express *TRAIL* + *GFP* in combination with magnetic nanoparticles (TRAIL + GFP + particles; referred to as cell-based microrobots) at a density of 35 × 10^4^ cells per insert in standard growth medium. Simultaneously, the bottom chambers were seeded with either healthy hAMSCs or human renal carcinoma cells (A498) at a density of 3.5 × 10^4^ cells per well. The viability of the bottom chamber cells was determined 72 hours after coculture. The Live/Dead Cell Imaging Kit (Invitrogen) was used to visualize the live (Ex/Em: 488/520 nm) and dead cells (Ex/Em: 528/617 nm) under a fluorescence microscope (Nikon Eclipse Ti-E). Moreover, cellular metabolic activity was quantified using the CellTiter-Glo Luminescent Cell Viability Assay (Promega) according to the manufacturer’s instructions. Luminescence values were measured in a 96-well black/clear bottom plate (Thermo Fisher Scientific) using a microplate reader (Tecan, Infinite M Plex).

### Magnetic actuation and steering of microrobots

Cell-based microrobots were magnetically actuated and steered using a custom-built five-coil electromagnetic setup integrated with an inverted microscope (Zeiss Axio Observer A1). Actuation was conducted within a microchannel on a glass substrate immersed in a cell culture medium under a uniform rotating magnetic field of 10 mT. The rotational frequency of the magnetic field was incrementally increased to modulate microrobot locomotion and to determine their step-out frequencies. The mean translational velocities of microrobots were calculated using a custom-developed MATLAB algorithm.

First, a single cell-based microrobot was magnetically controlled along a predefined trajectory by changing the direction of an applied 10-mT magnetic field at a frequency of 20 Hz. To evaluate the long-term magnetic actuation capability of the cell-based microrobots, the microrobots were detached from the cell culture plate and maintained at 37°C and 5% CO_2_. At defined time points (1 hours, 2 hours, and 3 hours), the microrobots were actuated under a magnetic field of 10 mT at 20 Hz for 5 min. Videos were recorded during actuation to quantify microrobot velocities using a custom MATLAB algorithm.

Next, the magnetic actuation and functional performance of the cell-based microrobots after repeated actuation over two consecutive days were investigated. Cell-based microrobots were collected on day 5, actuated at 10 mT and 20 Hz for 5 min, and subsequently reseeded into a 24-well plate. After overnight incubation, the culture medium was collected for measuring secreted TRAIL amounts using the TRAIL ELISA kit (BD Biosciences). This procedure was repeated for two consecutive days. Nonactuated but passaged cells were used as the control group.

Furthermore, the magnetic navigation capabilities of cell-based microrobots were evaluated using 3D tumor spheroids. Both the tumor spheroid and cell-based microrobots were placed into a microchannel filled with cell culture medium. A custom-built five-coil electromagnetic setup integrated with an inverted microscope (Zeiss Axio Observer A1) was used for magnetic actuation. A uniform rotating magnetic field with a strength of 10 mT guided the microrobots toward the tumor spheroid. Real-time video imaging was recorded to visualize and track their movement.

### Cellular viability after magnetic actuation

A rotating magnetic field of 10 mT at 20 Hz was applied to cell-based microrobots under static conditions for 5 min to evaluate the effects of magnetic actuation on cell viability after repeated actuation over two consecutive days. Following actuation, the cells were incubated in a humidified incubator at 37°C with 5% CO_2_. The following day, using the Live/Dead Cell Imaging Kit (Invitrogen) and fluorescence microscopy (Nikon Eclipse Ti-E), viability was evaluated. Moreover, cell viability was measured quantitatively by using the CellTiter-Glo Luminescent Cell Viability Assay (Promega), following the manufacturer’s instructions. Luminescence values were measured using a microplate reader (Tecan, Infinite M Plex).

### Assessment of 3D tumor spheroids death

A498 cells were seeded at a density of 5 × 10^3^ cells per well in Nunclon Sphera–treated 96-well plates with U-shaped bottoms (Thermo Fisher Scientific) and incubated at 37°C in a 5% CO_2_ atmosphere. The 3D tumor spheroids were formed by day 3 and used for further analysis. The A498 tumor spheroids were treated with (i) fresh medium, (ii) medium harvested from unmodified 293T cells, and (iii) medium harvested from cell-based microrobots for 24 hours. Following incubation, the tumor spheroids were stained with a Live/Dead Cell Imaging Kit (Invitrogen). The live (Ex/Em: 488/520 nm) and dead cells (Ex/Em: 528/617 nm) were visualized under a fluorescence microscope (Nikon Eclipse Ti-E). A498 tumor spheroids were also measured for ATP levels using the CellTiter-Glo 3D Cell Viability Assay (Promega). Briefly, an equal volume of CellTiter-Glo 3D reagent was added to the spheroids and mixed for 5 min for cell lysis, and the signal was stabilized for 25 min at room temperature. Luminescence values were then measured in a 96-well black/clear-bottom plate (Thermo Fisher Scientific) using a microplate reader (Tecan, Infinite M Plex).

### Statistical analysis

For each group, at least three replicate measurements were conducted. Numerical values are presented as the means ± standard deviation (SD). Statistical analysis was performed using Student’s *t* tests or one-way analysis of variance (ANOVA) with Tukey’s post hoc multiple-comparison tests. Significance is indicated as *P < 0.05, **P < 0.01, ***P < 0.001, and ****P < 0.0001, with *P* ≥ 0.05 considered not significant (n.s.).

## Supplementary Materials

### The PDF file includes:

Supplementary Methods Figs. S1 to S24 Tables S1 to S5 Legends for movies S1 to S5 References
