# Supplementary material for: Genetically engineered human cell–based microrobots for selective cancer cell death
Source: Sci Adv. 2026 Apr 29;12(18):eaea9831. doi: 10.1126/sciadv.aea9831 (PMC13127565; doi:10.1126/sciadv.aea9831)
Supplement: Supplementary file 1 — Supplementary Methods Figs. S1 to S24 Tables S1 to S5 Legends for movies S1 to S5 References [file sciadv.aea9831_sm.pdf]

Science Advances
AAAS

Supplementary Materials for

Genetically engineered human cell–based microrobots for selective cancer cell death

Nihal Olcay Dogan *et al.*

Corresponding author: Metin Sitti, msitti@ku.edu.tr

Sci. Adv. **12**, eaea9831 (2026)
DOI: 10.1126/sciadv.aea9831

The PDF file includes:

Supplementary Methods
Figs. S1 to S24
Tables S1 to S5
Legends for movies S1 to S5
References

Other Supplementary Material for this manuscript includes the following:

Movies S1 to S5

Methods

Cytotoxicity of magnetic Janus particles: To evaluate the biocompatibility of magnetic Janus particles, a black/clear-bottom 96-well plate (Corning) was seeded in triplicate with 293T cells at a density of 1×10^4 cells per well. Cells were allowed to adhere overnight. After 24 hours, cells were exposed to magnetic Janus particles at various concentrations (untreated, 0.05, 0.1, 0.2, and 0.4 mg/mL). CellTiter-Glo Luminescent Cell Viability Assay (Promega) was used to quantify cellular ATP levels, according to the manufacturer's protocol. A microplate reader (Tecan Infinite M Plex) was used to measure luminescence intensity. In parallel, cells were also stained with a Live/Dead Cell Imaging Kit (Invitrogen) according to the manufacturer's instructions. The live (Ex/Em: 488/520 nm) and dead cells (Ex/Em: 528/617 nm) were visualized under a fluorescent microscope (Nikon Eclipse Ti-E).

Dynamic light scattering (DLS) and zeta potential measurements: Size distribution and zeta potential of magnetic Janus particles (500 nm-diameter silica cores, half-coated with a 60 nm-thick FePt nanofilm) suspended in DMEM were measured using a Zetasizer Ultra (Malvern Panalytical).

Shear stability of cell-based microrobots and safety of detached particles: Cell-based microrobots were prepared as outlined in fig. S12. To assess the mechanical stability, adherent cell-based microrobots were first subjected to orbital shaking (Biozym shaker, 19-mm orbital diameter) in a 12-well plate containing 1 mL of medium (fluid height ~2.4-3.0 mm) at 150 rpm for 1 h. The cells were then detached using trypsin to generate a non-adherent cell suspension, which was subsequently exposed to orbital shaking under the same conditions (12-well plate, 1 mL medium, fluid height ~2.4-3.0 mm, 150 rpm) for an additional 30 min. The particle detachment was examined in both adherent cells, to model surface-associated shear experienced by cells anchored within tissues or vessel walls, and suspension cells, to capture the dynamic flow-like shear conditions that more closely mimic the mechanical forces acting on freely circulating cells.

The orbital shaking parameters applied in these experiments correspond to an estimated wall shear stress of ~10-20 dyn/cm^2, with higher local peaks near the well periphery (~20-30 dyn/cm^2) (*68*, *69*). These estimates are based on computational fluid dynamics (CFD) analysis of orbital shaking systems with comparable shaking speeds, fluid heights, and orbit diameters. The resulting

shear levels are consistent with the physiological shear stresses, including venous shear stresses (~1-6 dyn/cm^2) and arterial shear stresses (~10-20 dyn/cm^2) (*70*). After shear exposure, cells were collected and analyzed by flow cytometry. For each group, 10,000 events were collected and analyzed using FlowJo software (version 10.8.1). Particle detachment was quantified by measuring the percentage of GFP$^+$/Cy5$^+$ cells.

Next, BJ human fibroblasts and hAMSCs (human adipose-derived mesenchymal stem cells) were seeded into black, clear-bottom 96-well plates (Corning) at a density of 1×10^4 cells per well and allowed to adhere overnight. The following day, cells were treated with conditioned media collected separately from adhered cell-based microrobots (shaken for 1 hour or unshaken) and from suspended cell-based microrobots (shaken for 30 minutes or unshaken). After overnight incubation, cell viability was evaluated by quantifying ATP levels using the CellTiter-Glo Luminescent Cell Viability Assay (Promega) according to the manufacturer's instructions. Luminescence was measured using a Tecan Infinite M Plex microplate reader.

Metabolic activity measurements: Mitochondrial metabolic activity was measured using the Cell Proliferation Kit I (MTT) (Sigma-Aldrich). 293T cells were seeded at a density of 1×10^4 cells per well in 96-well plates and subjected to four different experimental conditions: (i) unmodified 293T cells, (ii) genetically modified 293T cells, (iii) unmodified 293T cells with magnetic Janus particles, and (iv) genetically modified 293T cells with magnetic Janus particles. On day 5, when the microrobots were fully prepared as described in fig. S12, cells were incubated with MTT reagent for 4 hours, followed by overnight incubation with the solubilization reagent based on the manufacturer's instructions. Absorbance values were measured at 570 nm with a reference wavelength of 650 nm using a Tecan Infinite M Plex microplate reader.

Optimization of duration for maximal transfection efficiency: To determine the maximal transfection efficiency, the effect of transfection duration was evaluated using the previously determined optimal 2:1 FuGENE 6 transfection reagent-to-DNA ratio. 293T cells were seeded into 6-well plates on Day 1 at a density of 3×10^5 cells per well in 2 mL of standard culture medium. On Day 2, transfection complexes were added, followed by overnight incubation. The next day, the transfection media were replaced with fresh media. Cells were harvested at 24, 48, and 72 h post-transfection to assess GFP fluorescence and TRAIL expression. The transfected cells were

initially imaged using a fluorescence microscope (Nikon Eclipse Ti-E) under the FITC channel (Ex/Em: 488/520 nm) to visualize GFP expression. Next, conditioned media from transfected cells were collected without being further concentrated and analyzed by human CD253 (TRAIL) ELISA kits (BD Biosciences), following the manufacturer's instructions. Absorbance measurements were made using a microplate reader (Tecan Infinite M Plex). Moreover, flow cytometry (BD LSRFortessa X-20, BD Biosciences) was used to quantify GFP^+ cells. The cells were detached from each group, fixed with 4% paraformaldehyde (v/v in DPBS) for 20 minutes, rinsed, and analyzed using a blue laser (Ex/Em: 488/525 nm). In each group, 10,000 events were collected and analyzed using FlowJo software (version 10.8.1). Based on the optimization results, a 2:1 transfection reagent-to-DNA ratio at 48 hours of post-transfection was selected for all subsequent experiments.

Determining the optimal therapeutic window for cell-based microrobots: For determining the optimal therapeutic window, 293T cells were seeded at 5×10^4 cells per well in 24-well plates on Day 1. On Day 2, transfection complexes were added and incubated overnight. After overnight incubation, the medium was replaced with fresh growth medium. On Day 4, corresponding to the 24 hours post-transfection time point, cells and culture supernatants were collected for further analysis. Then, particles were added to the remaining cultures, and incubation continued to enable subsequent time-point measurements. Cells and supernatants were collected at the following time points (48, 72, and 96 h post-transfection). GFP expression was quantified by flow cytometry to assess transfection efficiency, Cy5 signal was measured by flow cytometry to evaluate magnetic particle retention, and TRAIL secretion was measured in the collected supernatants using human CD253 ELISA kit (BD Biosciences).

Long-term TRAIL secretion: For the long-term TRAIL secretion experiment, 293T cells were seeded at 2×10^4 cells per well in 24-well plates. Beginning on Day 4 (fig. S12), corresponding to Day 1 post-transfection, and continuing through Day 10 (7 days post-transfection), culture supernatants were collected daily from the cell-based microrobots. Secreted TRAIL levels were quantified using a human CD253 (TRAIL) ELISA set from BD Biosciences, following the manufacturer's protocol. Absorbance was measured with a Tecan Infinite M Plex microplate reader.

***In vitro* immune activation analysis:** J774A.1 mouse macrophages were incubated with (i) natural growth medium as a control, (ii) magnetic Janus particles alone, (iii) 293T cell-based microrobots at a 1:1 macrophage-to-microrobot ratio, or (iv) bacterial lipopolysaccharides (LPS) (200 ng/mL) as a positive control. After 24 hours of incubation, culture supernatants were collected to assess cytokine secretion. Levels of TNF-α and IL-6 were quantified using mouse TNF-α and IL-6 ELISA sets (BD Biosciences). Absorbance was measured using a Tecan Infinite M Plex microplate reader.

Selective apoptosis in human cancer cell lines induced by cell-based microrobots: To evaluate the targeted cytotoxic potential of cell-based microrobots, a black/clear-bottom 96-well plate (Corning) was seeded in triplicate with human healthy cell lines—including human fibroblasts (BJ) and human adipose-derived mesenchymal stem cells (hAMSC)—at a density of 1×10^4 cells per well. Prior to treatment, the cells were allowed to adhere overnight to ensure optimal viability and attachment. After 24 hours, cells were exposed either to conditioned medium from unmodified 293T cells (containing 0 ng/mL TRAIL) or to conditioned medium from cell-based microrobots, which included an average of 220 ng/mL TRAIL measured by human CD253 TRAIL ELISA (BD Biosciences). The CellTiter-Glo Luminescent Cell Viability Assay (Promega) was used to quantify cellular ATP levels, which serve as a marker for metabolic activity and viability. A microplate reader (Tecan Infinite M Plex) was used to measure luminescence intensity. To provide qualitative insights, differential interference contrast (DIC) microscopy (Nikon Eclipse Ti-E inverted microscope) was used to examine cell morphology.

In a parallel investigation, human cancer cell lines—including colorectal carcinoma (HCT116), glioblastoma (T98G), renal adenocarcinoma (ACHN), and ovarian adenocarcinoma (ONCO-DG-1)—were similarly seeded in triplicate at 1×10^4 cells per well and incubated overnight for adherence. The next day, these cancer cells were treated for 24 hours with the conditioned medium (containing 0 ng/mL TRAIL) or with conditioned medium from cell-based microrobots, containing an average of 220 ng/mL TRAIL measured by human CD253 TRAIL ELISA (BD Biosciences). The CellTiter-Glo assay was used to quantify intracellular ATP concentrations post-treatment, and luminescence readings were obtained using the Tecan Infinite

M Plex microplate reader system. With the Nikon Eclipse Ti-E microscope, morphological changes in cancer cells were captured using DIC imaging.

Dose-response characterization of recombinant TRAIL in 2D and 3D cultures: We first determined IC_{50} values in 2D cell cultures. HCT116, T98G, ACHN, A498, and ONCO-DG-1 cells were seeded in triplicate at 1×10^4 cells per well in 96-well plates. The cells were either kept untreated or treated with increasing concentrations of recombinant superkiller TRAIL (Enzo Life Sciences), ranging from 0.3 ng/mL to 1000 ng/mL. The following day, the CellTiter-Glo Luminescent Cell Viability Assay (Promega) was used to measure cell viability, and a Tecan Infinite M Plex microplate reader was used to record luminescence.

We next determined IC_{50} values using 3D cultures of cell lines that form stable, compact spheroids. Spheroid formation was induced for 3 days on Nunclon Sphera-treated 96-well plates with U-shaped bottoms (Thermo Fisher Scientific) by seeding 5×10^3 cells per well and incubating them at 37°C in 5% CO_2. Spheroids were either left untreated or treated with recombinant superkiller TRAIL (Enzo Life Sciences) at concentrations ranging from 0.3 ng/mL to 1000 ng/mL. The CellTiter-Glo 3D assay (Promega) was used to test viability, and luminescence was measured with the Tecan Infinite M Plex microplate reader.

Dose-dependent cytotoxicity of TRAIL in ONCO-DG-1 cells: ONCO-DG-1 cancer cells were seeded in triplicate into black, clear-bottom 96-well plates (Corning) at a density of 1×10^4 cells per well and allowed to adhere overnight. The following day, cells were treated with commercial recombinant human TRAIL/TNFSF10 protein (R&D Systems) at varying concentrations (25, 50, 100, and 200 ng/mL). Cellular ATP levels, indicative of cell viability, were quantified using the CellTiter-Glo Luminescent Cell Viability Assay (Promega) according to the manufacturer’s protocol. Luminescence intensity was measured using a Tecan Infinite M Plex microplate reader.

Quantitative RT-PCR analysis of transfected 293T cells: 293T cell pellets were harvested after the transfection and stored at -80°C until further processing. Total RNA was extracted using the Nucleospin RNA kit (Macherey-Nagel, Germany) following the manufacturer’s instructions and subsequently stored at -80°C. For each sample, cDNA was synthesized from 1000 ng of total RNA. Quantitative RT-PCR was carried out using the SYBR Green Master Mix (Roche, Switzerland) on

a LightCycler 480 system (Roche, Switzerland). Relative gene expression levels were determined using the ΔΔCt method with GAPDH serving as the reference gene. Primers are listed in table S4.

Viral packaging and transduction of BJ fibroblasts: Viral packaging was carried out as previously described (*71*). In brief, 2.5×10^6 293T cells were plated in 10-cm dishes containing DMEM supplemented with 10% FBS and 1% Pen/Strep on day 0. On the following day, viral plasmid DNA (2.5 µg) was combined with the packaging plasmids (psPAX2, pUMVC (2.5 µg each), and VSV-G (0.25 µg)) and transfected into the cells using Fugene HD (Promega). The medium was replaced the next day. Viral supernatant was collected at 48 and 72 hours after transfection, passed through 0.45 µm filters, aliquoted, and stored at -80°C.

For viral transduction, target cells were plated at the desired density and infected the following day using viral supernatant supplemented with protamine sulfate (10 µg/mL). After overnight incubation, the medium was replaced with fresh growth medium. The transduced cells were then selected with puromycin (2 µg/mL) for three days. All lentiviral or retroviral vectors used in this study are listed in table S5.

Quantitative RT-PCR analysis of transduced BJ cells: BJ skin fibroblast cell pellets were harvested after transduction and stored at -80°C until further processing. Total RNA was extracted using the Nucleospin RNA kit (Macherey-Nagel, Germany) following the manufacturer's protocol and subsequently stored at -80°C. For each sample, cDNA was synthesized from 1000 ng of total RNA. Quantitative RT-PCR was carried out using the SYBR Green Master Mix (Roche, Switzerland) on a LightCycler 480 system (Roche, Switzerland). Relative gene expression levels were determined using the ΔΔCt method with GAPDH serving as the reference gene. Primers are listed in table S4.

Viability assessment with fibroblast-conditioned media: Cell viability was assessed using the CellTiter-Glo Luminescent Cell Viability Assay (Promega). A total of 1×10^4 cells per well were seeded into 96-well plates (Corning Costar; clear bottom, black-walled) and treated with conditioned media collected from transduced fibroblasts. For Tet-inducible vectors (pLIX-GFP and pLIX-TRAIL), conditioned media were harvested after 48 hours of doxycycline (Dox) treatment (0.1 µg/mL).

Supplementary Figures

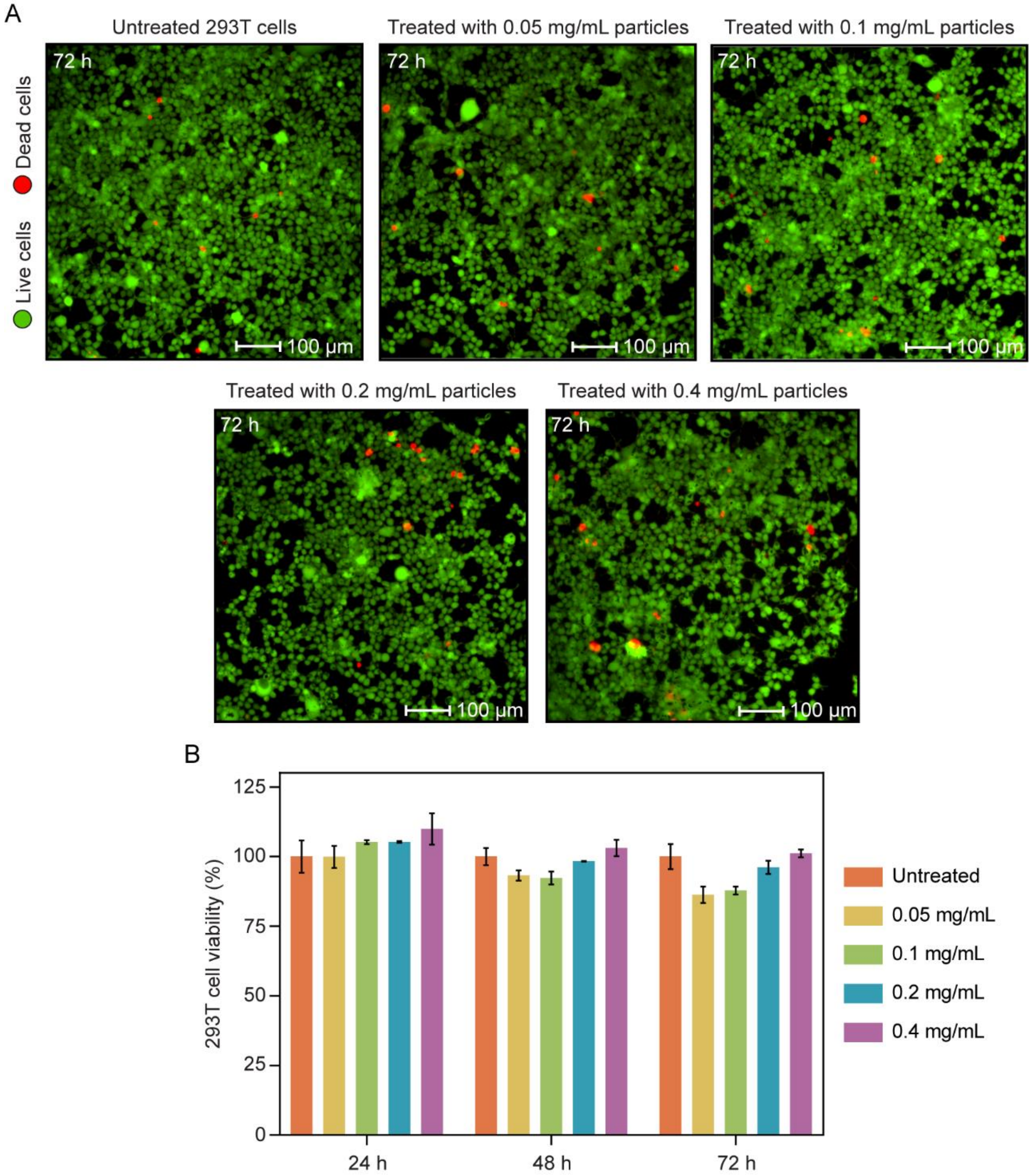

Fig. S1. Cytotoxicity of magnetic Janus particles on 293T cells. (A) Live/dead fluorescence imaging was performed after 72 h of incubation with magnetic Janus particles at concentrations of 0 mg/mL, 0.05 mg/mL, 0.1 mg/mL, 0.2 mg/mL, and 0.4 mg/mL to assess their biocompatibility. Images are displayed in two rows: untreated, 0.05, and 0.1 mg/mL (top row, left to right), and 0.2 and 0.4 mg/mL (bottom row, left to right). Fluorescence images of untreated 293T cells and 293T cells treated with magnetic Janus particles in all concentrations indicate that no cytotoxicity was observed, and most cells still had intact plasma membranes. Scale bars: 100 µm. **(B)** The percentage of viable 293T cells was quantified with the CellTiter-Glo assay after 24, 48, and 72

hours of treatment with magnetic Janus particles at concentrations of 0, 0.05, 0.1, 0.2, and 0.4 mg/mL. In all conditions, cell viability and proliferation remained similar to untreated controls. Data are presented as mean ± SD from n = 3 technical replicates.

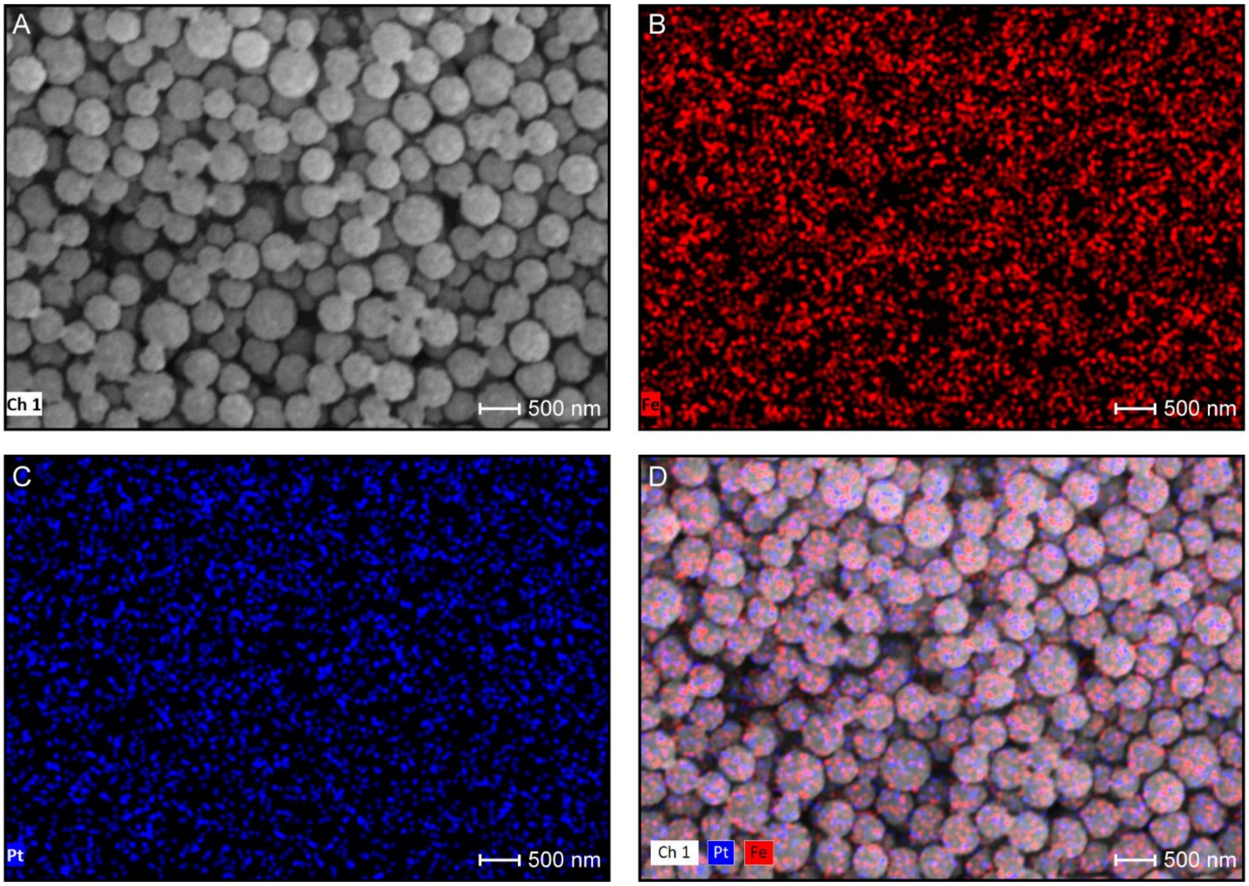

Fig. S2. SEM and EDX characterization of magnetic Janus particles. (A) SEM image of 500 nm-diameter silica beads coated with a 60 nm-thick FePt nanofilm. **(B)** EDX elemental map of Fe. **(C)** EDX elemental map of Pt. **(D)** Overlay of the SEM image and EDX maps, showing that Fe and Pt are homogeneously distributed on the surface of the particles. Scale bars: 500 nm.

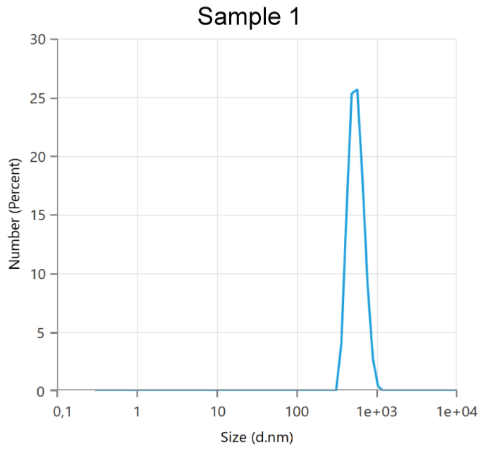

Name	Mean
Z-Average (nm)	825,8
Polydispersity Index (PI)	0,452
Peak 1 Mean by Intensity ordered by area (nm)	598,7
Peak 1 Area by Intensity ordered by area (%)	100

Name	Mean
Zeta Potential (mV)	-20,03
Conductivity (mS/cm)	16,63
Wall Zeta Potential (mV)	0
Quality Factor	1,834

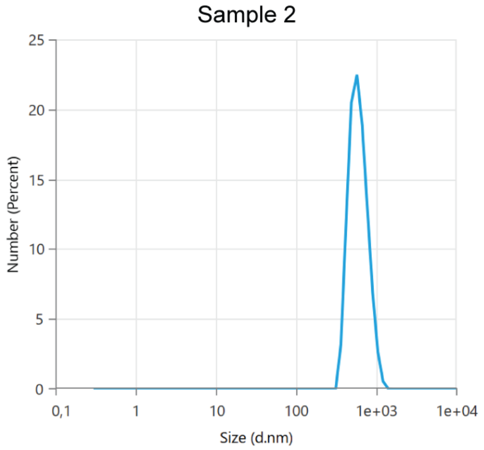

Name	Mean
Z-Average (nm)	757,3
Polydispersity Index (PI)	0,2963
Peak 1 Mean by Intensity ordered by area (nm)	665,1
Peak 1 Area by Intensity ordered by area (%)	100

Name	Mean
Zeta Potential (mV)	-18,67
Conductivity (mS/cm)	17,01
Wall Zeta Potential (mV)	0
Quality Factor	2,34

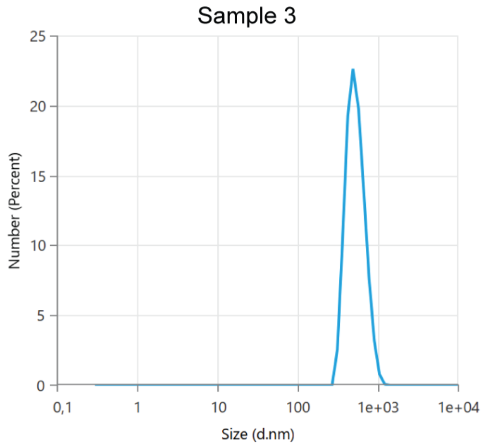

Name	Mean
Z-Average (nm)	707,3
Polydispersity Index (PI)	0,3361
Peak 1 Mean by Intensity ordered by area (nm)	594,1
Peak 1 Area by Intensity ordered by area (%)	100

Name	Mean
Zeta Potential (mV)	-19,9
Conductivity (mS/cm)	17,2
Wall Zeta Potential (mV)	0
Quality Factor	2,685

Fig. S3. DLS size distribution and zeta potential measurements of magnetic Janus particles dispersed in cell culture medium. Hydrodynamic size distributions demonstrated main peaks at 598.7 nm, 665.1 nm, and 594.1 nm, yielding an average diameter of 619 ± 40 nm. Similarly, the Z-average values were 825.8 nm, 757.3 nm, and 707.3 nm with an average of 763 ± 59 nm. These values are consistent with the expected size after FePt coating (~60 nm) of the original silica particles (0.4–0.6 µm; supplier's data). The obtained polydispersity index (PI) values range between 0.30 and 0.45, indicating moderate polydispersity. Accordingly, the measured zeta

potentials of -20.03 mV, -18.67 mV, and -19.9 mV with an average of -19.5 ± 0.8 mV indicate relatively stable dispersions in culture medium. Table S2 summarizes the data obtained from these three measurements.

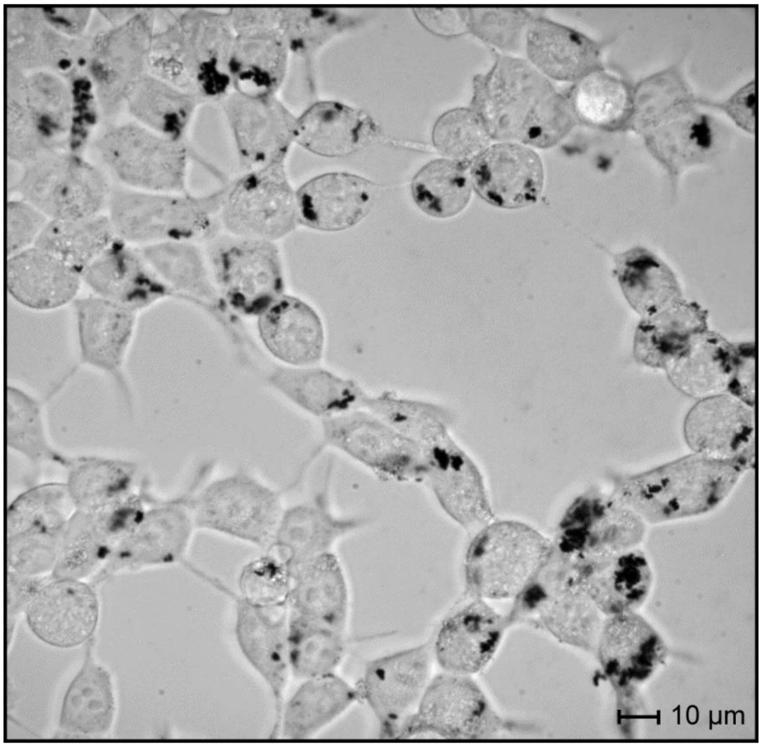

Fig. S4. Phase-contrast microscopy images of 293T cells incubated with magnetic Janus particles overnight, without any post-processing steps (*e.g.*, no washing). The black spherical objects represent the particles, which are observed in close contact with the cell surface, rather than being freely distributed in the culture medium or deposited on the culture plate surface. Scale bar: 10 μm.

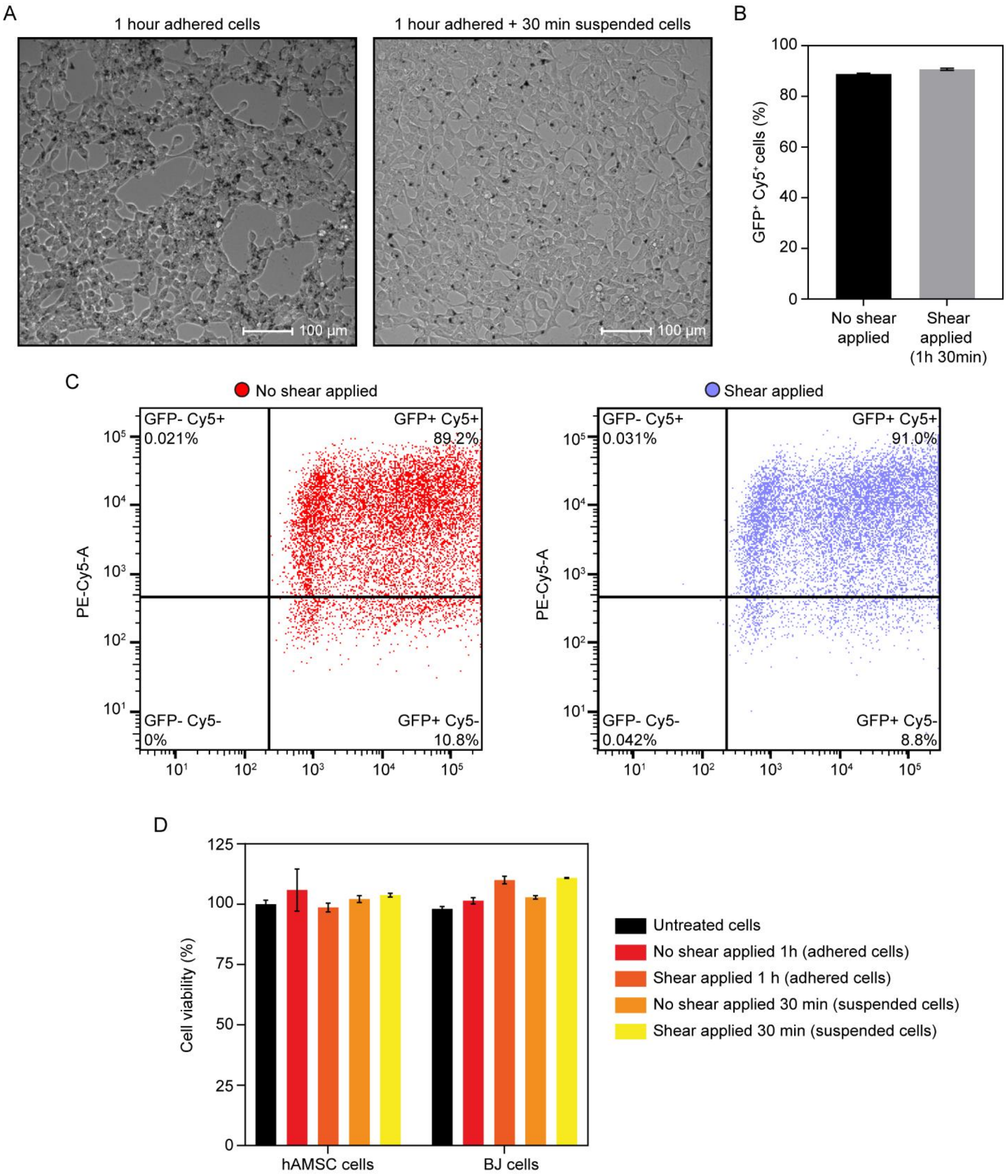

Fig. S5. Shear stability studies of cell-based microrobots. (A) DIC images of cell-based microrobots exposed to shear: (i) 1 h orbital shear with attached cells, followed by (ii) 30 min shear applied to trypsinized, detached, floating cells, which were imaged after overnight incubation. Scale bars: 100 µm. **(B)** Flow cytometry analysis of GFP^+ $Cy5^+$ cells shows no significant difference between shear-applied and non-shear control groups. Data were presented

as mean ± SD from n = 3 technical replicates. **(C)** Representative flow cytometry quadrant gating for shear-applied and non-shear control samples. **(D)** Viability of hAMSC and BJ healthy cells treated with conditioned medium collected from shear-applied cell-based microrobots. No decrease in cell viability was observed in both cell lines in all conditions. Data were presented as mean ± SD from n = 3 technical replicates.

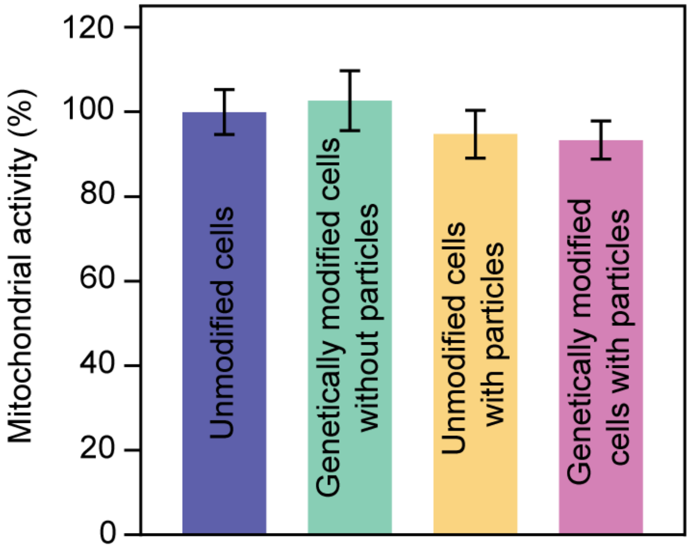

Fig. S6. Assessment of mitochondrial metabolic activity using the MTT assay. MTT assay results showing mitochondrial metabolic activity of (i) unmodified 293T cells, (ii) genetically modified cells without particles, (iii) unmodified cells with particles, and (iv) genetically modified cells with particles. Values are presented as a percentage relative to unmodified cells. Data were presented as mean ± SD from n = 3 independent biological replicates, each with three technical measurements.

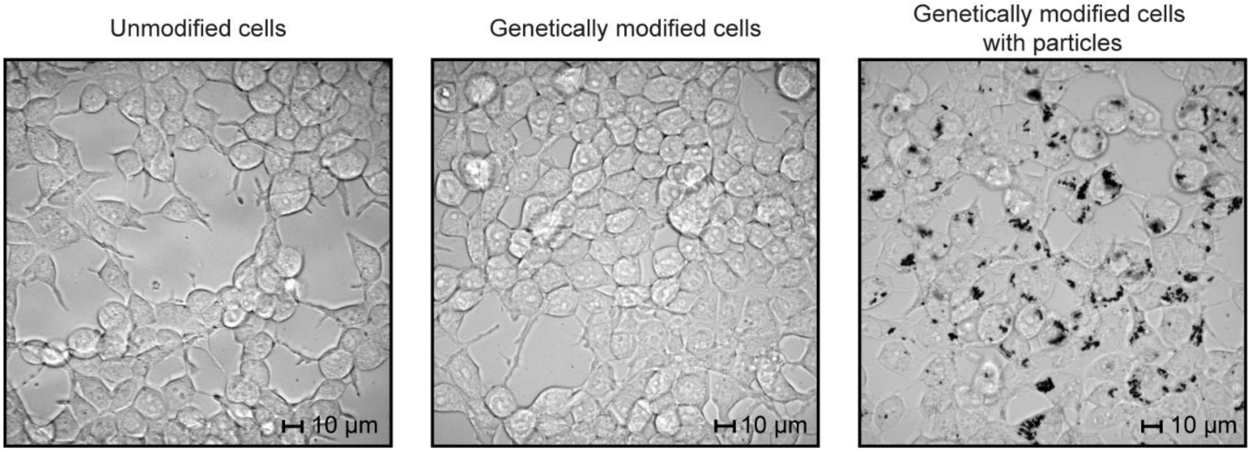

Fig. S7. Phase-contrast microscopy images of 293T cells. Phase-contrast microscopy imaging of (i) unmodified 293T cells, (ii) genetically modified 293T cells, and (iii) genetically modified 293T cells conjugated with magnetic Janus particles. To enable better visual comparison between experimental groups, the unmodified cells and genetically modified cells with particles used in Fig. 2D were also applied here. No noticeable morphological changes were observed across the groups, consistent with the viability assay results. Scale bars: 10 μm.

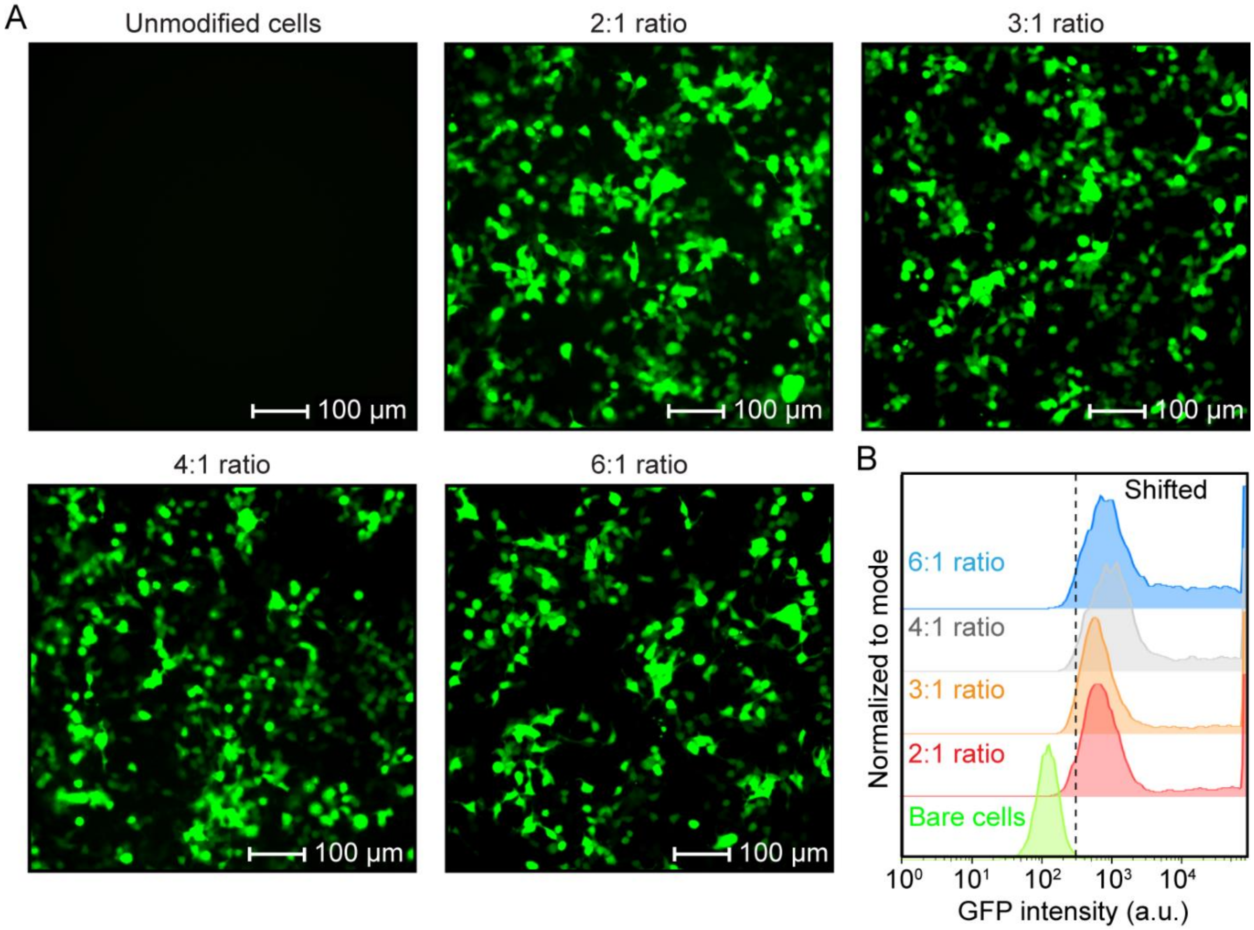

Fig. S8. Optimization of the transfection reagent-to-DNA ratio based on GFP expression. 293T cells were transfected with different transfection reagent-to-DNA ratios (2:1, 3:1, 4:1, and 6:1). **(A)** Representative fluorescence microscopy images were captured for all groups. As expected, unmodified 293T cells did not express GFP, whereas all transfected groups exhibited high GFP expression. For better visual comparison between different experimental groups, the same 2:1 ratio used in Fig. 2A was applied here as well. Scale bars: 100 μm. **(B)** Flow cytometry analysis further confirmed enhanced GFP expression across all transfected groups compared to the control cells.

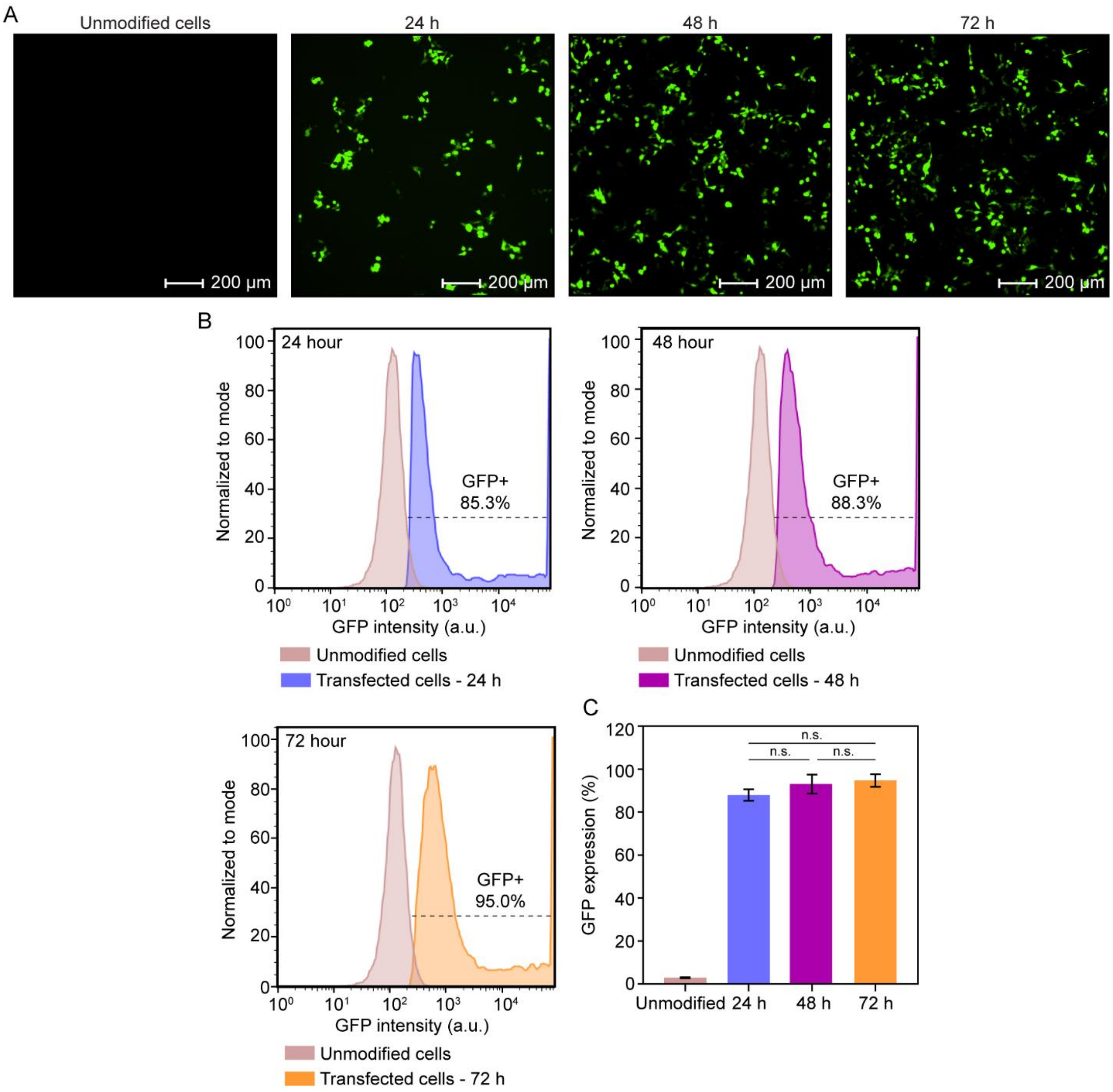

Fig. S9. Time-dependent optimization of transfection efficiency based on GFP expression. **(A)** To determine the optimal duration for GFP expression, the transfection efficiency of 293T cells was evaluated at three different time points (24 h, 48 h, and 72 h). Representative fluorescence microscopy images were obtained for each group. As expected, unmodified 293T cells showed no GFP expression, whereas all transfected groups exhibited strong GFP signals. Scale bars: 200 µm. **(B)** A quantitative flow cytometry analysis was performed to evaluate the GFP expression over time in a single experimental group. **(C)** The percentage of GFP-expressing cells was determined as 88.0 ± 2.7%, 93.2 ± 4.4%, and 94.8 ± 3.0% at 24, 48, and 72 hours, respectively. Data are presented as mean ± SD, n = 3 technical replicates. One-way ANOVA with Tukey's post-hoc test; n.s. indicates $p \geq 0.05$. Compared to the unmodified group, all time points (24, 48, and 72 h) showed a significant difference (****$p < 0.0001$).

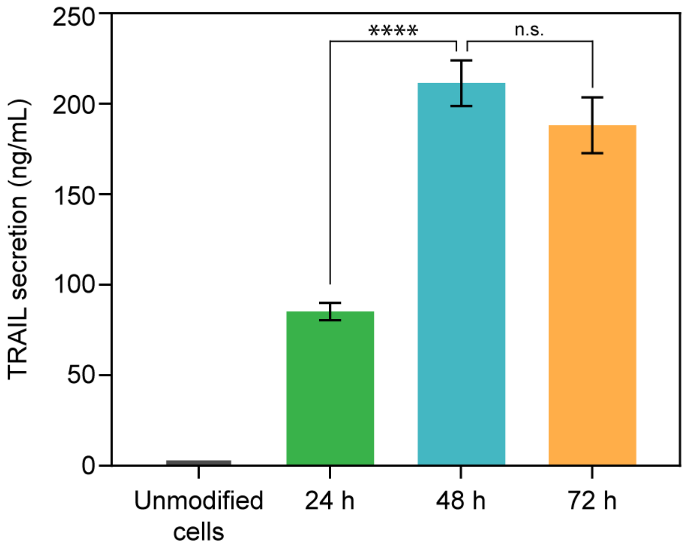

Fig. S10. Time-dependent optimization of transfection efficiency based on TRAIL expression. Even though optimizing GFP expression provided insight into transfection efficiency, this study primarily focused on TRAIL expression. To evaluate this, TRAIL expression levels were quantified at different time points (24, 48, and 72 hours) using ELISA. Since the highest expression was observed at 48 hours, all subsequent experiments were conducted at this time point. Data are presented as mean ± SD, n = 3 technical replicates. One-way ANOVA with Tukey's post-hoc test; n.s. indicates $p \geq 0.05$, and ****$p < 0.0001$.

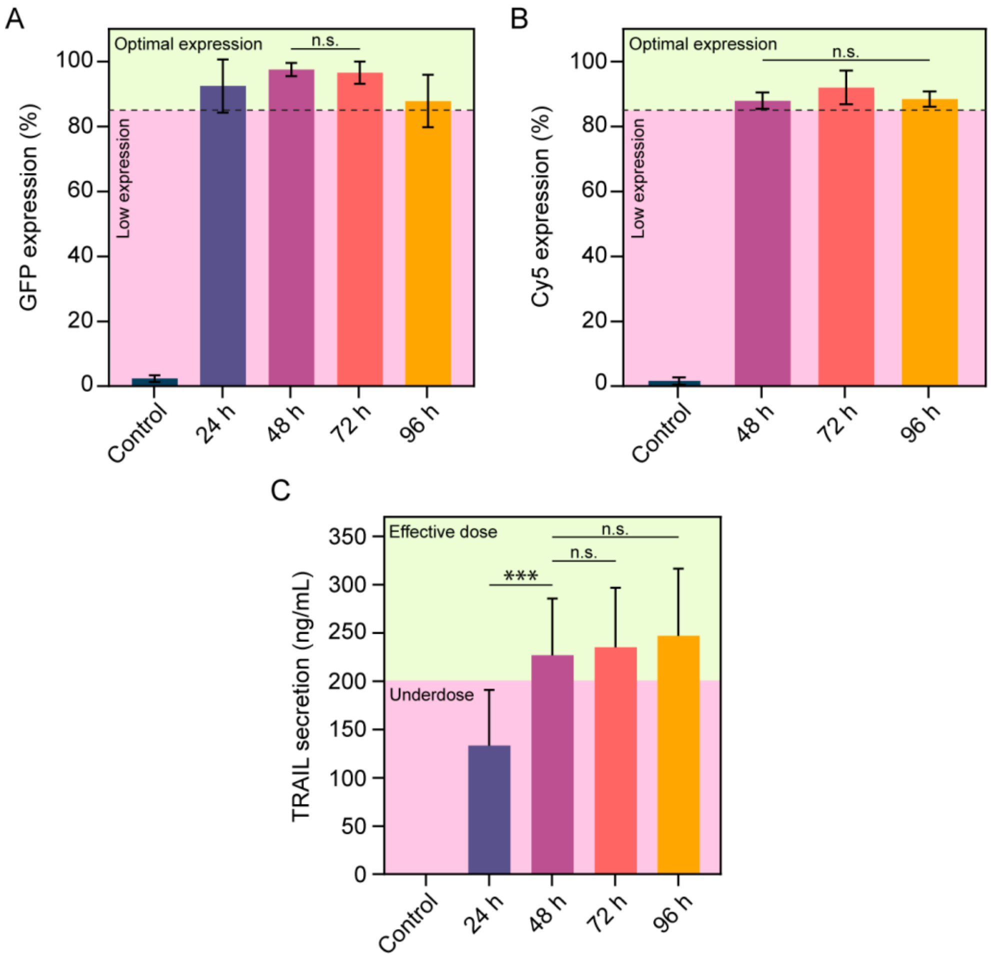

Fig. S11. Determining the optimal therapeutic window for cell-based microrobots. Time-dependent measurements at 24, 48, 72, and 96 h post-transfection were performed to evaluate **(A)** transfection efficiency indicated by GFP fluorescence (flow cytometry), **(B)** magnetic particle retention indicated by Cy5 fluorescence (flow cytometry), **(C)** TRAIL secretion quantified by ELISA. Line graphs illustrate changes at each time point to define the optimal therapeutic window, ranging from 48 h to 96 h post-transfection, which corresponds to peak TRAIL secretion (>200 ng/mL), high particle retention (>85%), and high transfection efficiency (>85%). Based on these results, 48 h post-transfection was selected for subsequent experiments as the optimal experimental time point, since the 24 h time point did not yield an effective TRAIL secretion level, while later time points showed no significant increases in TRAIL secretion, particle retention, or GFP expression. Data were presented as mean ± SD from n = 3 independent biological replicates, each with three technical measurements. One-way ANOVA with Tukey's post-hoc test; n.s. indicates $p \geq 0.05$, and $***p < 0.001$.

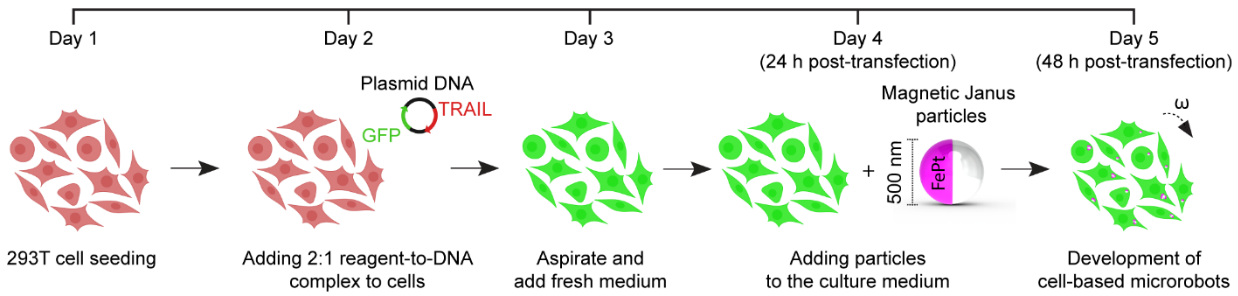

Fig. S12. Timeline of the cell-based microrobots fabrication process. Cell-based microrobots were developed through a five-day protocol. On Day 1, 293T cells were seeded into culture plates under standard conditions. On Day 2, the transfection reagent-to-DNA complex at a 2:1 ratio was prepared and added dropwise to the culture medium. On Day 3, the transfection medium was carefully aspirated and replaced with a fresh medium. On Day 4, the culture medium from the transfected 293T cells was collected, and magnetic Janus particles were added at a final concentration of 0.2 mg/mL. The particle-containing medium was then reapplied to the cells to promote particle-cell conjugation. As of Day 5, the cell-based microrobots were fully assembled and ready for experimentation.

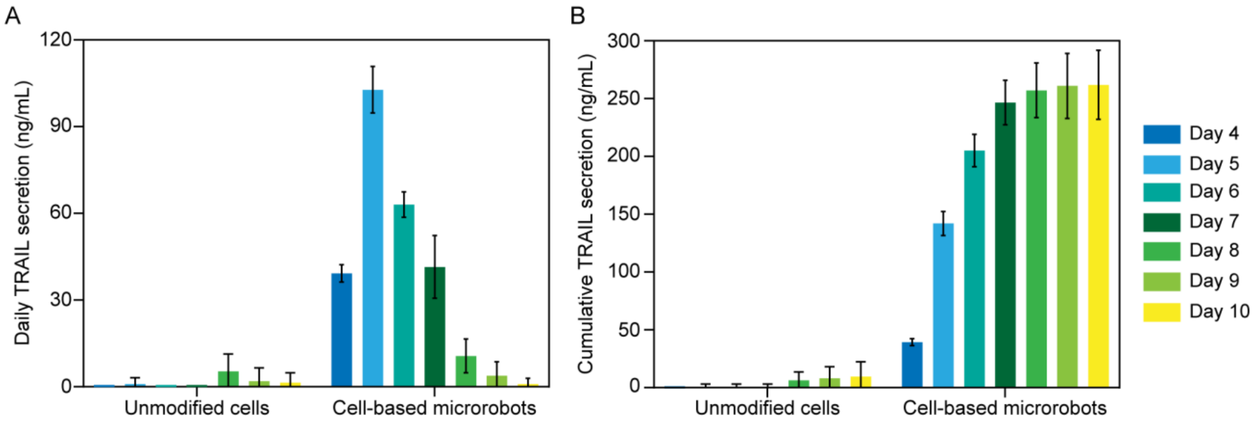

Fig. S13. TRAIL secretion over several days. TRAIL secretion was quantified by collecting culture medium from the cell-based microrobots each day. Measurements began on Day 4 (24 hr post-transfection) to allow sufficient time for TRAIL expression, and continued daily until Day 10. **(A)** Daily TRAIL secretion, **(B)** cumulative TRAIL secretion. Data were presented as mean ± SD from n = 3 independent biological replicates, each with two technical measurements.

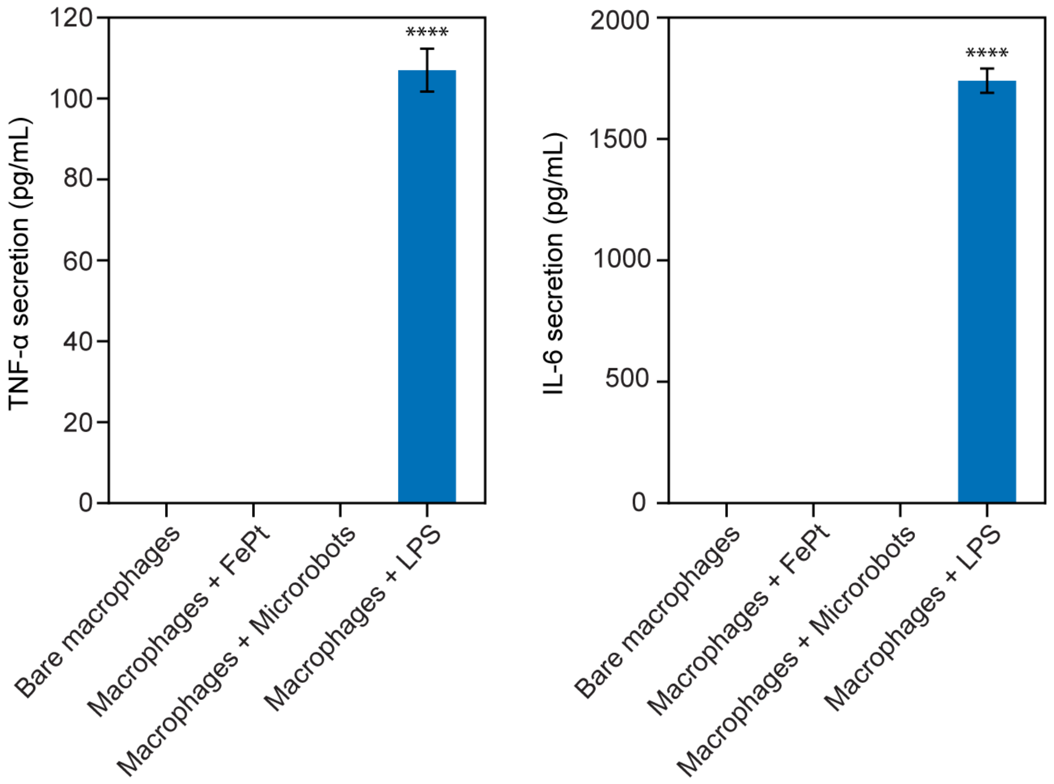

Fig. S14. Co-culture of macrophages with cell-based microrobots did not trigger macrophage activation. J774A.1 mouse macrophages were co-cultured with TRAIL-expressing 293T cell-based microrobots. After overnight co-culture, the culture medium was collected and analyzed for mouse TNF-α and mouse IL-6 secretion. No increase in either cytokine was observed compared with the bare macrophages, whereas the LPS-treated positive control showed a strong cytokine response in both assays. These results indicate that the cell-based microrobots did not induce macrophage activation, suggesting a biosafety profile and no detectable pro-inflammatory or immunostimulatory response under *in vitro* conditions. This supports the immunological safety of the system and provides an important basis for future *in vivo* studies. One-way ANOVA with Tukey's post-hoc test; ****$p < 0.0001$.

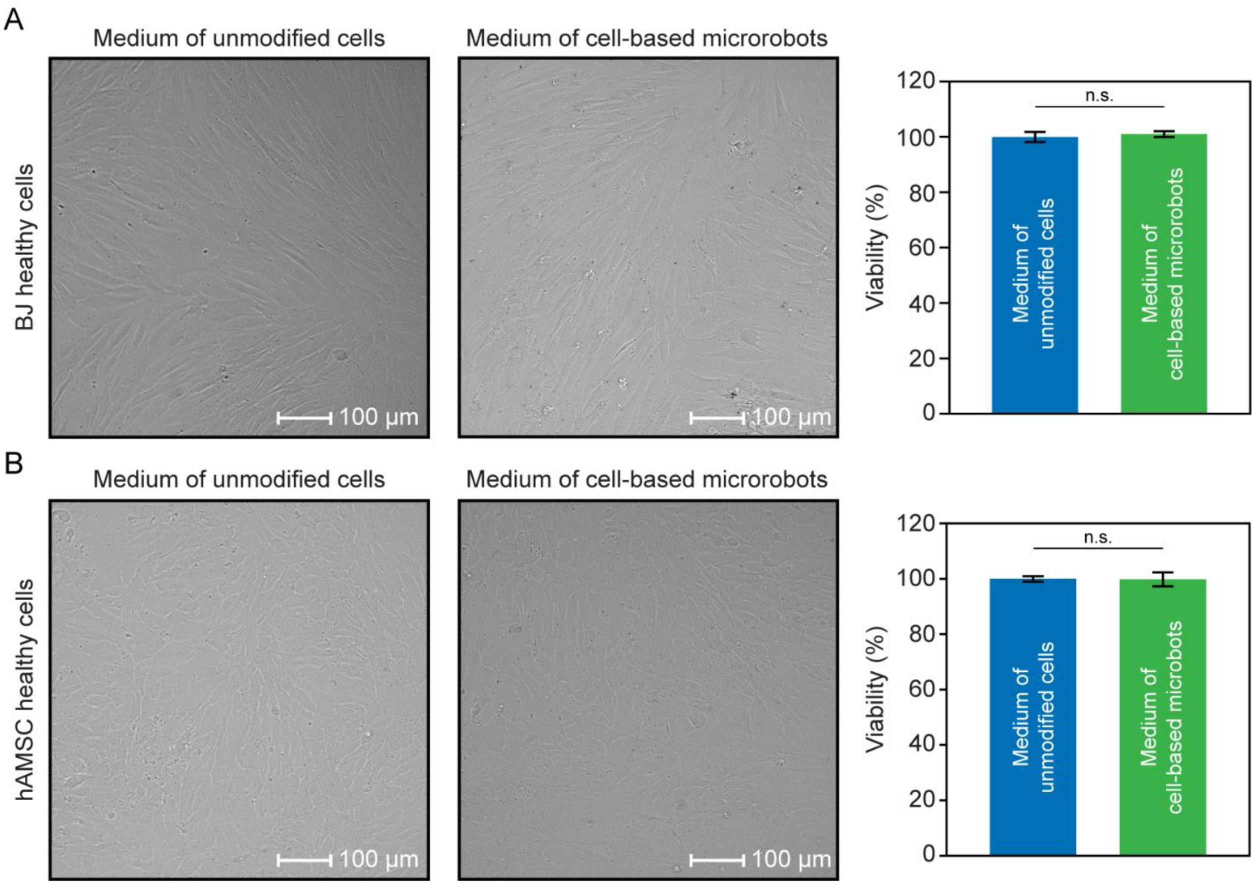

Fig. S15. Selective cancer-killing properties of cell-based microrobots: Evaluation with healthy cells. Cell-based microrobots were evaluated for their potential cytotoxicity on healthy cells, including **(A)** healthy BJ human fibroblasts and **(B)** healthy hAMSC human adipose-derived mesenchymal stem cells. Representative DIC images were acquired after 24 h incubation with the conditioned medium from unmodified 293T cells or cell-based microrobots. No notable morphological changes were observed. Cell viability was further assessed by quantifying intracellular ATP levels using the CellTiter-Glo assay. Results showed no significant decrease in ATP levels compared to the control group, indicating that the conditioned medium from cell-based microrobots did not exert cytotoxic effects on healthy BJ and hAMSC cells. The microrobot-conditioned medium contained an average of 220 ng/mL TRAIL, whereas medium from unmodified 293T cells contained no detectable TRAIL. (Data are presented as mean ± SD, $n = 3$ technical replicates; n.s. indicates $p \geq 0.05$, Student's t-test). Scale bars: 100 µm.

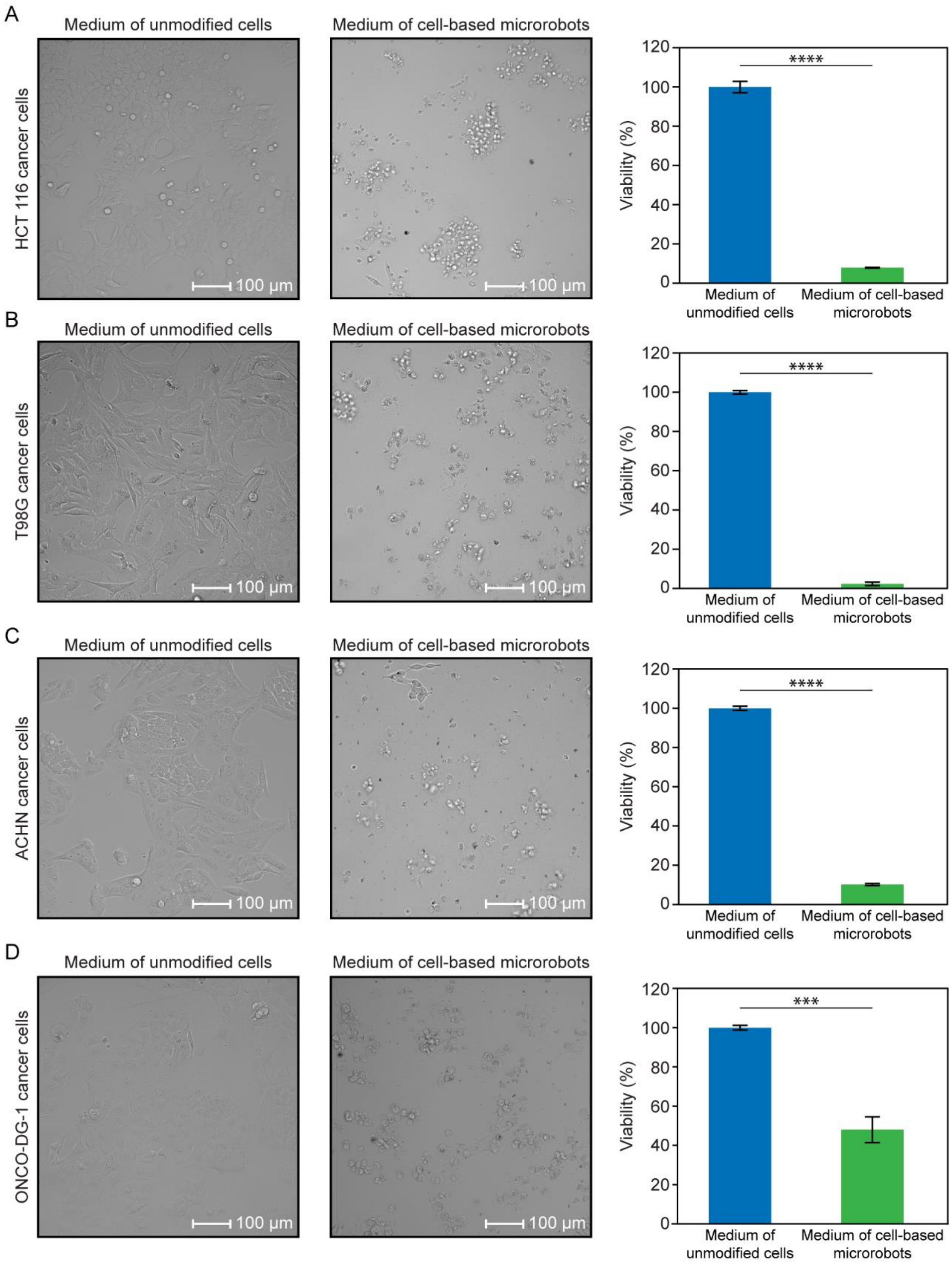

Fig. S16. Selective cancer-killing properties of cell-based microrobots: Evaluation with cancer cells. The cytotoxic effects of cell-based microrobots were further assessed using various human cancer cell lines. **(A)** HCT116 colon cancer cells showed extensive cell death and disrupted morphology after 24 h incubation with a microrobot-conditioned medium, where the cell viability was reduced to 7.9 ± 0.2% as measured by ATP quantification. **(B–D)** Similar results were

observed in T98G glioblastoma cells, ACHN renal carcinoma cells, and ONCO-DG-1 ovarian cancer cells, all of which exhibited apparent morphological alterations. T98G and ACHN cells demonstrated significantly decreased viabilities of 2.4 ± 0.8% and 10.2 ± 0.6%, respectively, while ONCO-DG-1 cells showed a moderate reduction to 48.0 ± 6.6%. These results indicate potent and selective cytotoxic effects of the cell-based microrobots on cancer cells and suggest that different cancer cell types exhibit varying sensitivity to TRAIL-mediated killing. The microrobot-conditioned medium contained an average of 220 ng/mL TRAIL, whereas medium from unmodified 293T cells contained no detectable TRAIL. Scale bars: 100 µm. Data are presented as mean ± SD, n = 3 technical replicates; ***$p < 0.001$, and ****$p < 0.0001$, Student's t-test. One-way ANOVA with Tukey's post-hoc test showed that ONCO-DG-1 cells were significantly more resistant to TRAIL than ACHN, HCT116, and T98G cells (****$p < 0.0001$), while no significant differences were observed among ACHN, HCT116, and T98G.

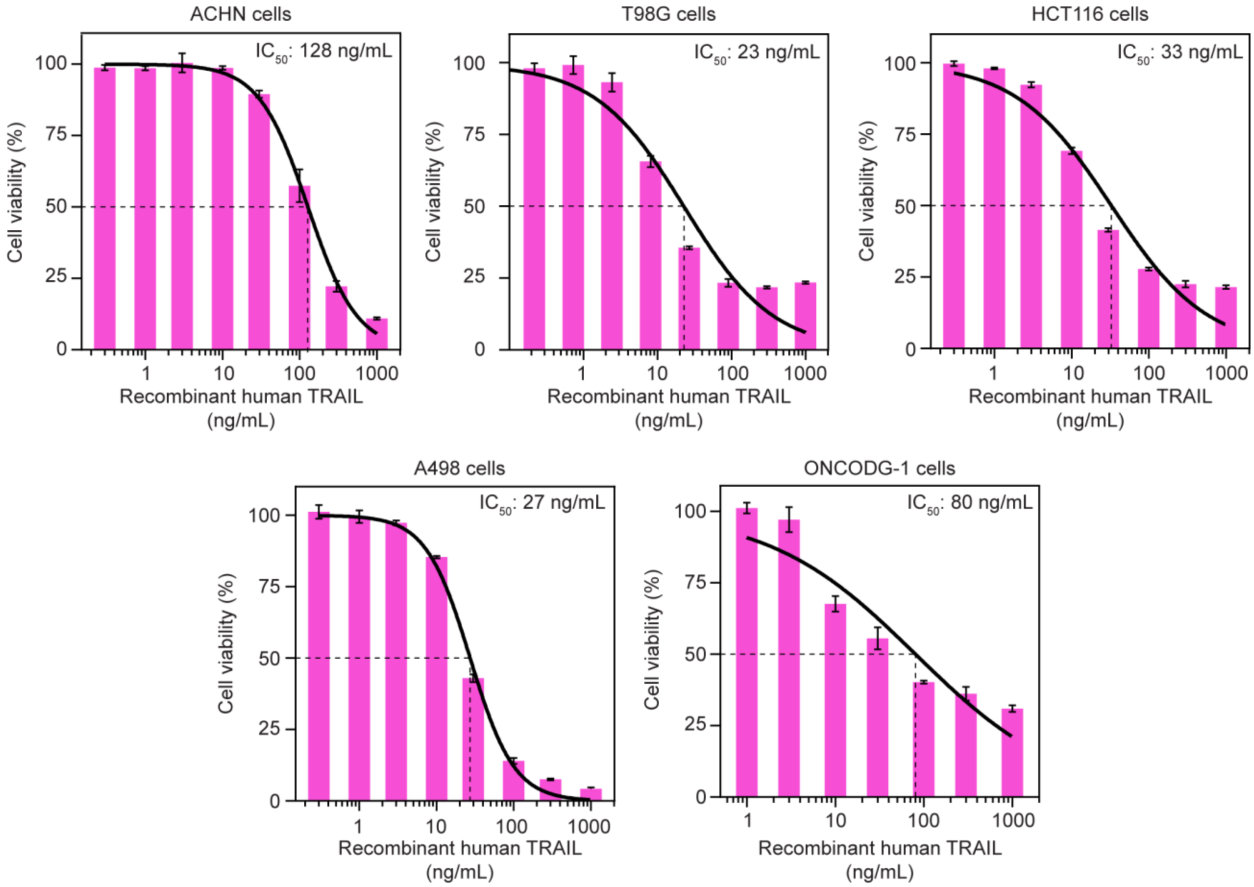

Fig. S17. IC_{50} dose-response curves of recombinant human TRAIL in 2D cell cultures. ACHN, T98G, HCT116, A498, and ONCO-DG-1 cells were treated with serial concentrations of recombinant human TRAIL ranging from 0.3 ng/mL to 1000 ng/mL on a logarithmic scale. Cell viabilities were determined using the CellTiter-Glo assay. Dose-response curves were generated by plotting the inhibitor concentration (recombinant TRAIL) against the normalized response (cell viability). Data were fitted using a nonlinear regression model with a variable slope in GraphPad Prism. IC_{50} values were defined as the concentrations of recombinant TRAIL that reduced cell viability to 50%. Data were presented as mean ± SD from n = 3 technical replicates.

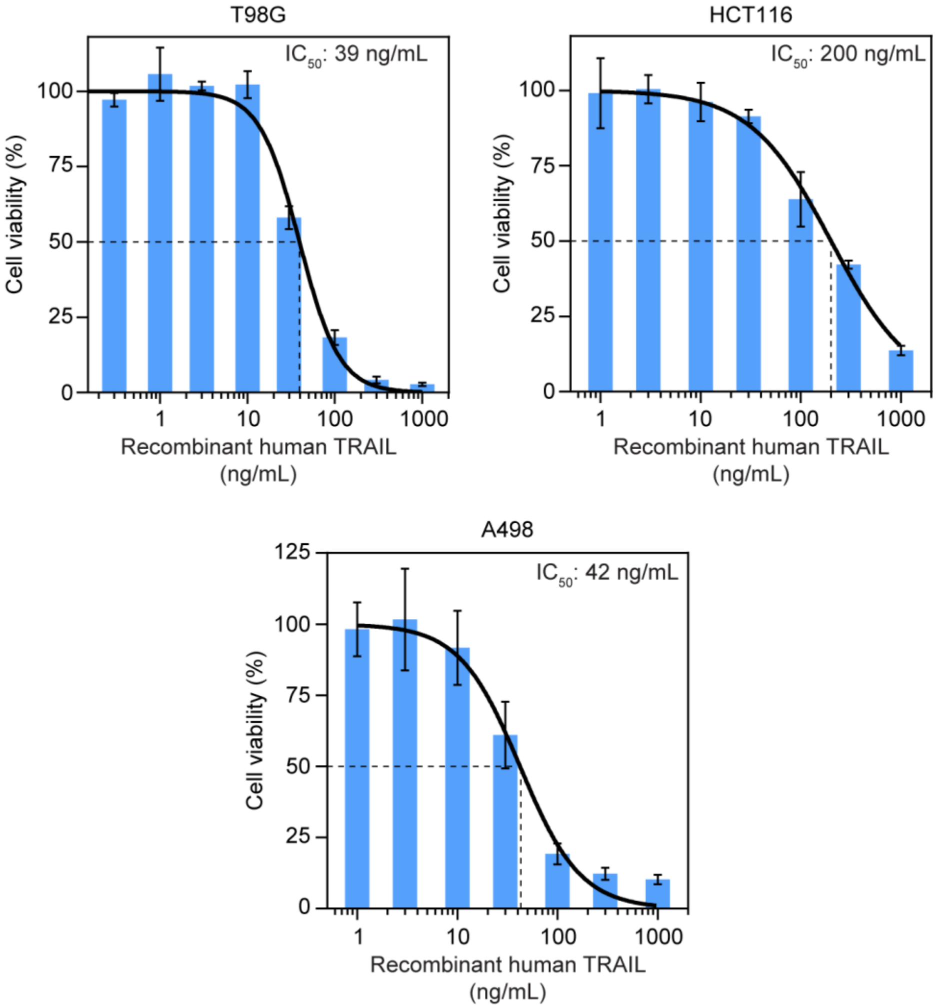

Fig. S18. IC_{50} dose-response curves of recombinant human TRAIL in 3D cell cultures. T98G, HCT116, and A498 spheroids were treated with serial concentrations of recombinant human TRAIL ranging from 0.3 ng/mL to 1000 ng/mL on a logarithmic scale. Cell viabilities were determined using the CellTiter-Glo 3D assay. Dose-response curves were generated by plotting the inhibitor concentration (recombinant TRAIL) against the normalized response (cell viability). Data were fitted using a nonlinear regression model with a variable slope in GraphPad Prism. IC_{50} values were defined as the concentrations of recombinant TRAIL that reduced the viability of 3D tumor spheroids to 50%. Data were presented as mean ± SD from n = 3 technical replicates.

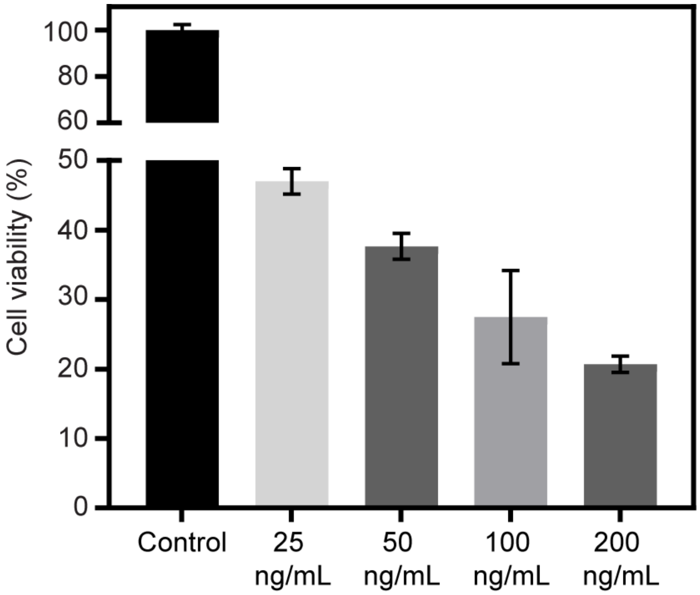

Fig. S19. Increased concentrations of recombinant TRAIL sensitize semi-resistant ONCO-DG-1 cancer cells. Increasing the commercial recombinant TRAIL concentration from 25 ng/mL to 200 ng/mL results in enhanced cytotoxicity, reducing cell viability from 47.0 ± 1.8% to 20.7 ± 1.2%. Data were presented as mean ± SD from n = 3 technical replicates.

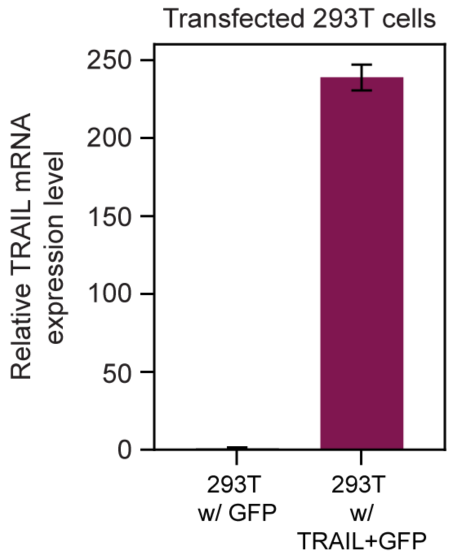

Fig. S20. qPCR analysis of TRAIL mRNA expression in 293T cells. 293T cells transfected with TRAIL+GFP showed an approximately 239-fold increase in TRAIL mRNA expression relative to GFP-modified control cells. Data were presented as mean ± SD from n = 3 technical replicates.

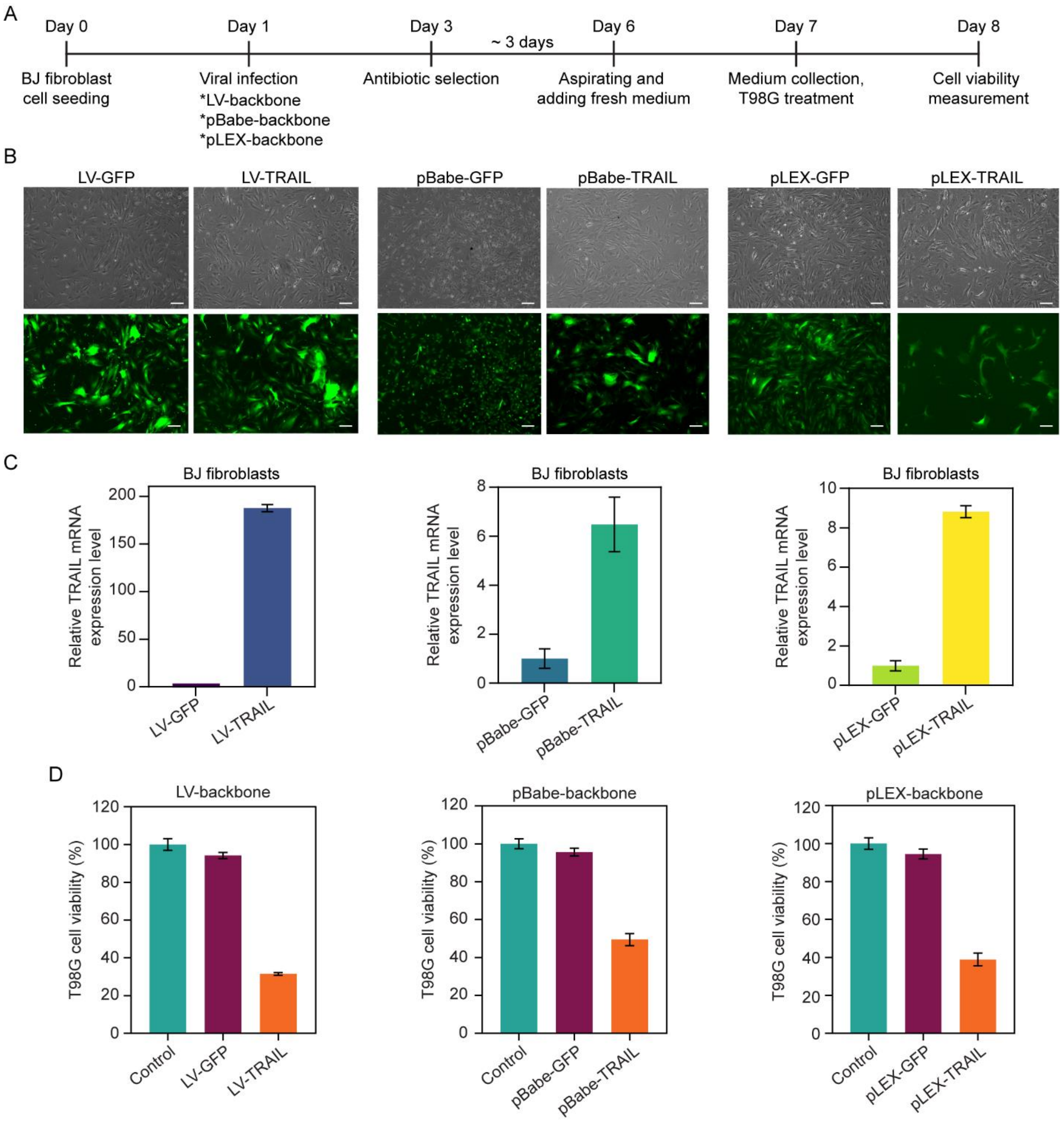

Fig. S21. Human fibroblasts transduced with the TRAIL gene using different vector backbones. (A) Experimental timeline for generating TRAIL-expressing human BJ fibroblasts via viral transduction and assessing the effect of their secreted TRAIL on the viability of T98G cells. **(B)** DIC and fluorescence images of BJ fibroblasts transduced with LV-GFP, LV-TRAIL, pBabe-GFP, pBabe-TRAIL, pLEX-GFP, and pLEX-TRAIL plasmid DNAs. Scale bars: 100 µm. **(C)** Quantification of TRAIL mRNA expression by qPCR. Relative to their corresponding GFP controls, TRAIL expression increased approximately 187-fold with LV-TRAIL, 6-fold with pBabe-TRAIL, and 9-fold with pLEX-TRAIL. Data were presented as mean ± SD from n = 3

technical replicates. **(D)** Viability of T98G glioblastoma cells cultured with conditioned medium collected from TRAIL-expressing BJ fibroblasts. T98G viability was reduced to 31.5 ± 0.7% with LV-TRAIL-transduced fibroblasts, 49.4 ± 3.2% with pBabe-TRAIL-transduced fibroblasts, and 38.9 ± 3.4% with pLEX-TRAIL-transduced fibroblasts. Data were presented as mean ± SD from n = 3 technical replicates.

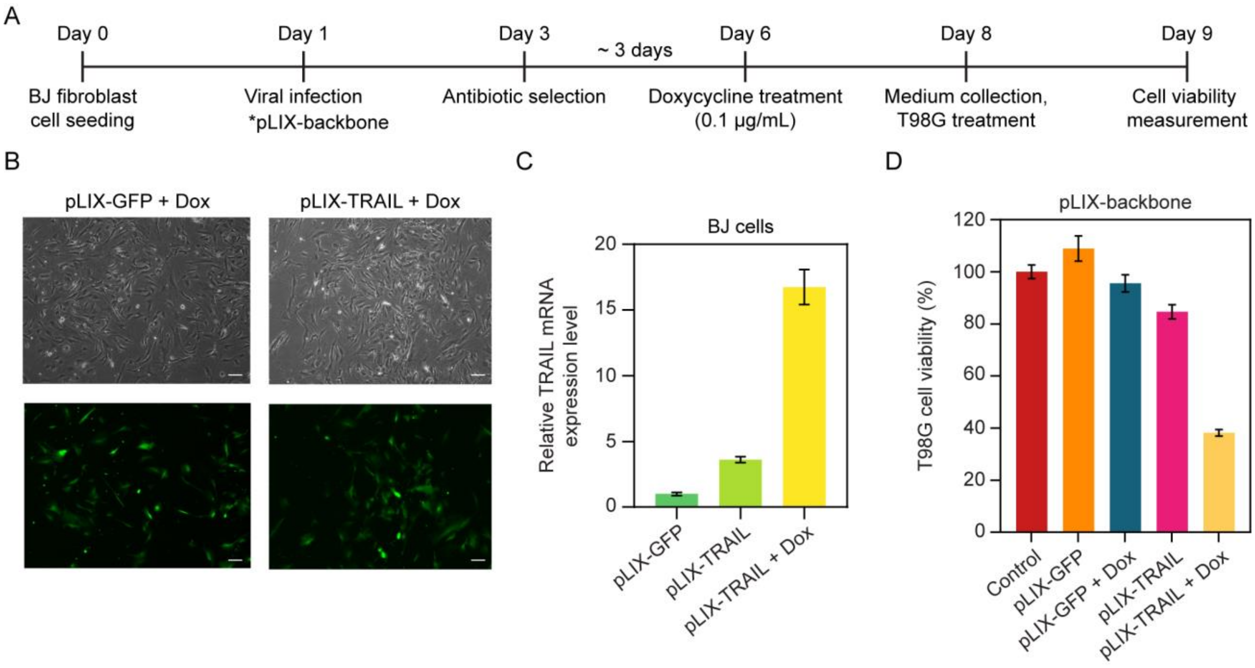

Fig. S22. Controlled TRAIL expression in human fibroblasts using a doxycycline-inducible pLIX-TRAIL backbone. (A) Experimental timeline for generating inducible TRAIL-expressing human BJ fibroblasts via viral transduction with the pLIX-TRAIL backbone and evaluating the effect of their secreted TRAIL on the viability of T98G cells. **(B)** DIC and fluorescence images of BJ fibroblasts transduced with the pLIX-GFP and pLIX-TRAIL plasmid following doxycycline induction. Scale bars: 100 μm. **(C)** Quantification of TRAIL mRNA expression by qPCR. Relative to the corresponding GFP controls, TRAIL expression increased approximately 17-fold in fibroblasts transduced with pLIX-TRAIL + Dox. Data were presented as mean ± SD from n = 3 technical replicates. **(D)** Viability of T98G cells cultured with conditioned medium collected from TRAIL-expressing BJ fibroblasts. In the presence of doxycycline (pLIX-TRAIL+Dox), T98G viability decreased to 38.2 ± 1.3%, whereas without doxycycline induction (pLIX-TRAIL), viability remained at 84.6 ± 2.7%. Data were presented as mean ± SD from n = 3 technical replicates.

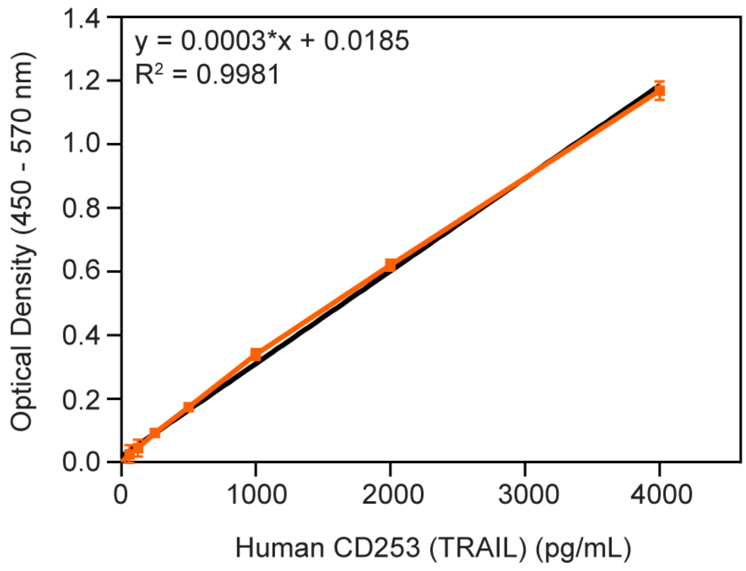

Fig. S23. Representative human CD253/TRAIL ELISA standard curve. Linear regression was used to generate the standard curve, yielding the equation y = 0.0003x + 0.0185 with an R^2 value of 0.9981 for the TRAIL ELISA assay. Samples were analyzed at dilution factors from 1:30 to 1:150 to ensure measurements fell within the linear detection range. Data were presented as mean ± SD from n = 3 technical replicates.

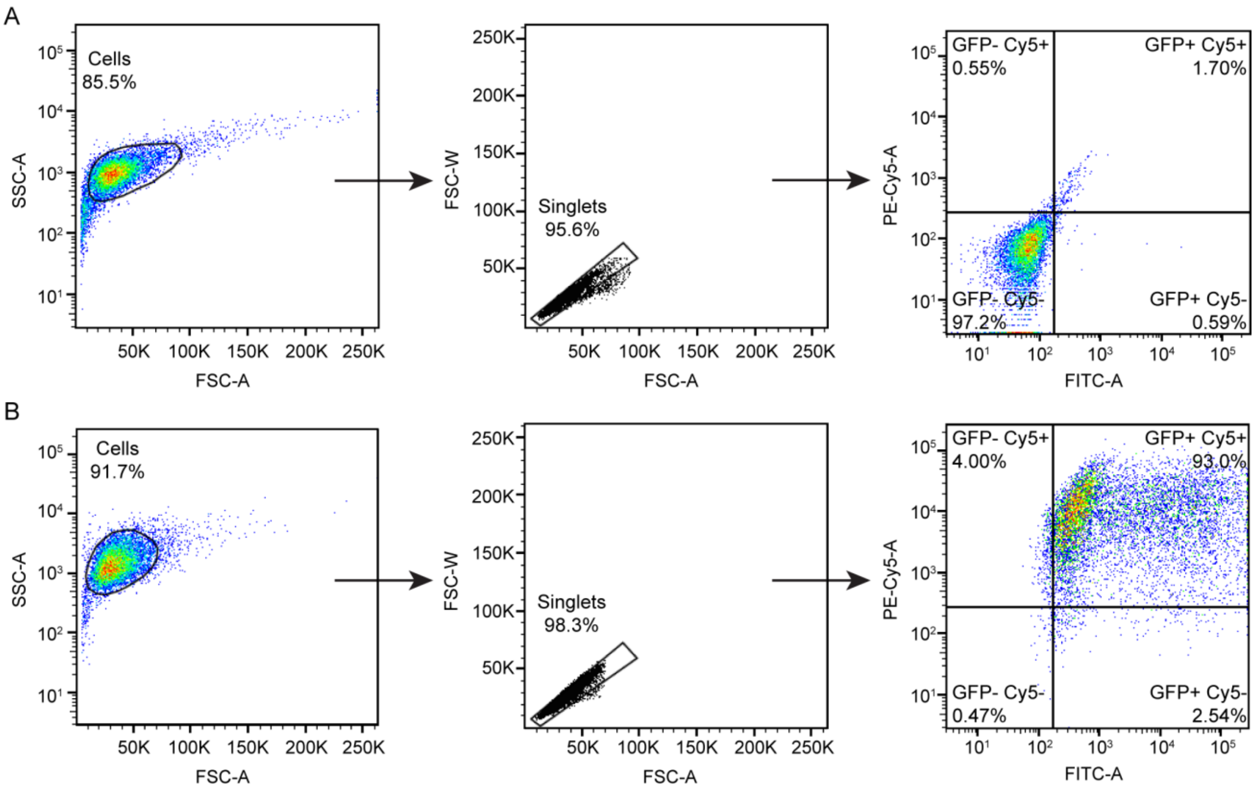

Fig. S24. Flow cytometry gating strategy. (A) Unmodified 293T cells, and **(B)** Cell-based microrobots. Cells were first gated using SSC-A (y-axis) vs. FSC-A (x-axis) to define the main population. Singlets were then selected using FSC-W (y-axis) vs. FSC-A (x-axis) to exclude doublets. Finally, GFP^+ and $Cy5^+$ cells were identified within the singlet population using quadrant gating.

Supplementary Tables

Table S1. EDX elemental composition of magnetic Janus particles. Elemental analysis showed atomic percentages of Fe (56.57 ± 1.04 at.%) and Pt (43.43 ± 2.98 at.%), indicating a near-equiatomic composition on the particle surface.

Element	Atomic number	Mass fraction (%)	Atomic fraction (%)	Absolute error (%)
Fe	26	13.16	56.57	1.04
Pt	78	35.31	43.43	2.98

Table S2. DLS and zeta potential measurements of magnetic Janus particles. Measurements were performed in culture medium, repeated in triplicate.

Sample number	Z-average (nm)	Polydispersity index (PDI)	Mean peak by intensity (nm)	Zeta potential (mV)
1	825.8	0.452	598.7	-20.03
2	757.3	0.2963	665.1	-18.67
3	707.3	0.3361	594.1	-19.9

Table S3. Transfection reagent-to-DNA ratio optimization parameters. Parameters used to optimize the transfection reagent-to-DNA ratios in a single well of a 6-well plate.

	Ratios of Transfection Reagent to DNA			
	**2:1**	**3:1**	**4:1**	**6:1**
Volume of FuGENE 6 transfection reagent	4 µL	6 µL	8 µL	12 µL
DNA amount	2 µg	2 µg	2 µg	2 µg
Final medium volume	100 µL	100 µL	100 µL	100 µL

Table S4. List of primers used in the study.

Gene	Sequence (5’→3’)	
TRAIL	F	TGCGTGCTGATCGTGATCTTC
	R	GCTCGTTGGTAAAGTACACGTA
GAPDH	F	AGCCACATCGCTCAGACAC
	R	GCCCAATACGACCAAATCC

Table S5. List of lentiviral and retroviral vectors used in this study.

Vectors	Cat. No
pUMVC	Addgene Plasmid #8449
psPAX2	Addgene Plasmid #12260
pCMV-VSV-G	Addgene Plasmid #8454
pLenti-GFP	Bagci-Onder, 2011.
pLenti-TRAIL	Bagci-Onder, 2011.
pBABE-GFP	Addgene Plasmid #10668
pBABE-TRAIL	Cloned for unpublished work.
pLEX-GFP	Cloned for unpublished work.
pLEX-TRAIL	Cloned for unpublished work.
pLIX-GFP	Ozyerli-Goknar, 2019
pLIX-TRAIL	Ozyerli-Goknar, 2019

Supplementary Videos

Movie S1. Magnetic actuation and steering of a single cell-based microrobot at 60 Hz frequency.

Movie S2. Magnetic actuation and steering of a single cell-based microrobot.

Movie S3. Magnetic actuation and steering of a swarm of cell-based microrobots.

Movie S4. Long-term magnetic actuation and steering of cell-based microrobot.

Movie S5. Magnetic guidance of cell-based microrobots towards a 3D tumor spheroid.

REFERENCES

1. M. Sitti, *Mobile Microrobotics* (MIT Press, 2017).

2. B. J. Nelson, I. K. Kaliakatsos, J. J. Abbott, Microrobots for minimally invasive medicine. *Annu. Rev. Biomed. Eng.* **12**, 55–85 (2010).

3. C. K. Schmidt, M. Medina-Sánchez, R. J. Edmondson, O. G. Schmidt, Engineering microrobots for targeted cancer therapies from a medical perspective. *Nat. Commun.* **11**, 5618 (2020).

4. V. Iacovacci, E. Diller, D. Ahmed, A. Menciassi, Medical microrobots. *Annu. Rev. Biomed. Eng.* **26**, 561–591 (2024).

5. S. Palagi, P. Fischer, Bioinspired microrobots. *Nat. Rev. Mater.* **3**, 113–124 (2018).

6. A. Del Campo Fonseca, C. Glück, J. Droux, Y. Ferry, C. Frei, S. Wegener, B. Weber, M. El Amki, D. Ahmed, Ultrasound trapping and navigation of microrobots in the mouse brain vasculature. *Nat. Commun.* **14**, 5889 (2023).

7. H. Han, X. Ma, W. Deng, J. Zhang, S. Tang, O. S. Pak, L. Zhu, E. Criado-Hidalgo, C. Gong, E. Karshalev, J. Yoo, M. Y. Ann Liu, C. Wang, H. K. Shen, P. N. Patel, C. L. Hays, P. J. Gunnarson, L. Li, Y. Zhang, J. O. Dabiri, L. V. Wang, M. G. Shapiro, D. Wu, Q. Zhou, J. R. Greer, W. Gao, Imaging-guided bioresorbable acoustic hydrogel microrobots. *Sci. Robot.* **9**, eadp3593 (2024).

8. J. Choi, J. Hwang, J. y. Kim, H. Choi, Recent progress in magnetically actuated microrobots for targeted delivery of therapeutic agents. *Adv. Healthc. Mater.* **10**, e2001596 (2021).

9. X. Ju, C. Chen, C. M. Oral, S. Sevim, R. Golestanian, M. Sun, N. Bouzari, X. Lin, M. Urso, J. S. Nam, Y. Cho, X. Peng, F. C. Landers, S. Yang, A. Adibi, N. Taz, R. Wittkowski, D. Ahmed, W. Wang, V. Magdanz, M. Medina-Sánchez, M. Guix, N. Bari, B. Behkam, R. Kapral, Y. Huang, J. Tang, B. Wang, K. Morozov, A. Leshansky, S. A. Abbasi, H. Choi, S. Ghosh, B. B. Fernandes, G. Battaglia, P. Fischer, A. Ghosh, B. J. Sánchez, A. Escarpa, Q. Martinet, J. Palacci, E. Lauga, J. Moran, M. A. Ramos-Docampo, B. Städler, R. S. H. Restrepo, G. Yossifon, J. D. Nicholas, J.

Ignés-Mullol, J. Puigmartí-Luis, Y. Liu, L. D. Zarzar, C. W. Shields IV, L. Li, S. Li, X. Ma, D. H. Gracias, O. Velev, S. Sánchez, M. J. Esplandiu, J. Simmchen, A. Lobosco, S. Misra, Z. Wu, J. Li, A. Kuhn, A. Nourhani, T. Maric, Z. Xiong, A. Aghakhani, Y. Mei, Y. Tu, F. Peng, E. Diller, M. S. Sakar, A. Sen, J. Law, Y. Sun, A. Pena-Francesch, K. Villa, H. Li, D. E. Fan, K. Liang, T. J. Huang, X.-Z. Chen, S. Tang, X. Zhang, J. Cui, H. Wang, W. Gao, V. K. Bandari, O. G. Schmidt, X. Wu, J. Guan, M. Sitti, B. J. Nelson, S. Pané, L. Zhang, H. Shahsavan, Q. He, I.-D. Kim, J. Wang, M. Pumera, Technology roadmap of micro/nanorobots. *ACS Nano* **19**, 24174–24334 (2025).

10. F. C. Landers, L. Hertle, V. Pustovalov, D. Sivakumaran, C. M. Oral, O. Brinkmann, K. Meiners, P. Theiler, V. Gantenbein, A. Veciana, M. Mattmann, S. Riss, S. Gervasoni, C. Chautems, H. Ye, S. Sevim, A. D. Flouris, J. Puigmartí-Luis, T. S. Mayor, P. Alves, T. Lühmann, X. Chen, N. Ochsenbein, U. Moehrlen, T. Schubert, Z. Kulcsar, P. Gruber, M. Weisskopf, Q. Boehler, S. Pané, B. J. Nelson, Clinically ready magnetic microrobots for targeted therapies. *Science* **390**, 710–715 (2025).

11. P. Wrede, E. Remlova, Y. Chen, X. L. Deán-Ben, M. Sitti, D. Razansky, Synergistic integration of materials in medical microrobots for advanced imaging and actuation. *Nat. Rev. Mater.* **10**, 888–906 (2025).

12. N. O. Dogan, E. Suadiye, P. Wrede, J. Lazovic, C. B. Dayan, R. H. Soon, A. Aghakhani, G. Richter, M. Sitti, Immune cell-based microrobots for remote magnetic actuation, antitumor activity, and medical imaging. *Adv. Healthc. Mater.* **13**, e2400711 (2024).

13. H. Ceylan, N. O. Dogan, I. C. Yasa, M. N. Musaoglu, Z. U. Kulali, M. Sitti, 3D printed personalized magnetic micromachines from patient blood–derived biomaterials. *Sci. Adv.* **7**, eabh0273 (2021).

14. B. Wang, J. Shen, C. Huang, Z. Ye, J. He, X. Wu, Z. Guo, L. Zhang, T. Xu, Magnetically driven biohybrid blood hydrogel fibres for personalized intracranial tumour therapy under fluoroscopic tracking. *Nat. Biomed. Eng.* **9**, 1471–1485 (2025).

15. N. O. Dogan, H. Ceylan, E. Suadiye, D. Sheehan, A. Aydin, I. C. Yasa, A. M. Wild, G. Richter, M. Sitti, Remotely guided immunobots engaged in anti-tumorigenic phenotypes for targeted cancer immunotherapy. *Small* **18**, 2204016 (2022).

16. H. Lutz, S. Hu, P.-U. Dinh, K. Cheng, Cells and cell derivatives as drug carriers for targeted delivery. *Med. Drug. Discov.* **3**, 100014 (2019).

17. Y. Elani, Interfacing living and synthetic cells as an emerging frontier in synthetic biology. *Angew. Chem. Int. Ed. Engl.* **133**, 5662–5671 (2021).

18. H. Zhang, Z. Li, C. Gao, X. Fan, Y. Pang, T. Li, Z. Wu, H. Xie, Q. He, Dual-responsive biohybrid neutrobots for active target delivery. *Sci. Robot.* **6**, eaaz9519 (2021).

19. V. D. Nguyen, H.-K. Min, H. Y. Kim, J. Han, Y. H. Choi, C.-S. Kim, J.-O. Park, E. Choi, Primary macrophage-based microrobots: An effective tumor therapy in vivo by dual-targeting function and near-infrared-triggered drug release. *ACS Nano* **15**, 8492–8506 (2021).

20. E. Totter, E. von Einsiedel, L. Regazzoni, S. Schuerle, Paving the way for bacteria-based drug delivery: Biohybrid microrobots emerging from microrobotics and synthetic biology. *Adv. Drug Deliv. Rev.*, **221**, 115577 (2025).

21. N. B. Day, C. R. Orear, A. C. Velazquez-Albino, H. J. Good, A. Melnyk, C. M. Rinaldi-Ramos, C. W. Shields IV, Magnetic cellular backpacks for spatial targeting, imaging, and immunotherapy. *ACS Appl. Bio. Mater.* **7**, 4843–4855 (2024).

22. Y. Dai, X. Bai, L. Jia, H. Sun, Y. Feng, L. Wang, C. Zhang, Y. Chen, Y. Ji, D. Zhang, H. Chen, L. Feng, Precise control of customized macrophage cell robot for targeted therapy of solid tumors with minimal invasion. *Small* **17**, e2103986 (2021).

23. C. W. Shields IV, Biohybrid microrobots for enhancing adoptive cell transfers. *Acc. Mater. Res.* **4**, 566–569 (2023).

24. X. Tang, Y. Yang, M. Zheng, T. Yin, G. Huang, Z. Lai, B. Zhang, Z. Chen, T. Xu, T. Ma, H. Pan, L. Cai, Magnetic-acoustic sequentially actuated CAR T cell microrobots for precision navigation and in situ antitumor immunoactivation. *Adv. Mater.* **35**, e2211509 (2023).

25. V. A. Webster-Wood, M. Guix, N. W. Xu, B. Behkam, H. Sato, D. Sarkar, S. Sanchez, M. Shimizu, K. K. Parker, Biohybrid robots: Recent progress, challenges, and perspectives. *Bioinspir. Biomim.* **18**, 015001 (2022).

26. S. A. Rosenberg, Lymphocytes as a living drug for cancer. *Science* **385**, 25–26 (2024).

27. A. W. Rankin, B. B. Duncan, C. Allen, S. K. Silbert, N. N. Shah, Evolving strategies for addressing CAR T-cell toxicities. *Cancer Metastasis Rev.* **44**, 17 (2025).

28. S. Du, J. Yan, Y. Xue, Y. Zhong, Y. Dong, Adoptive cell therapy for cancer treatment. *Exploration* **3**, 20210058 (2023).

29. B. Wu, B. Zhang, B. Li, H. Wu, M. Jiang, Cold and hot tumors: From molecular mechanisms to targeted therapy. *Signal Transduct. Target. Ther.* **9**, 274 (2024).

30. A. Fus-Kujawa, P. Prus, K. Bajdak-Rusinek, P. Teper, K. Gawron, A. Kowalczuk, A. L. Sieron, An overview of methods and tools for transfection of eukaryotic cells in vitro. *Front. Bioeng. Biotechnol.* **9**, 701031 (2021).

31. R. C. Sterner, R. M. Sterner, CAR-T cell therapy: Current limitations and potential strategies. *Blood Cancer J.* **11**, 69 (2021).

32. A. C. Uscanga-Palomeque, A. K. Chávez-Escamilla, C. A. Alvizo-Báez, S. Saavedra-Alonso, L. D. Terrazas-Armendáriz, R. S. Tamez-Guerra, C. Rodríguez-Padilla, J. M. Alcocer-González, CAR-T cell therapy: From the shop to cancer therapy. *Int. J. Mol. Sci.* **24**, 15688 (2023).

33. H. Lin, X. Yang, S. Ye, L. Huang, W. Mu, Antigen escape in CAR-T cell therapy: Mechanisms and overcoming strategies. *Biomed. Pharmacother.* **178**, 117252 (2024).

34. J. N. Brudno, J. N. Kochenderfer, Toxicities of chimeric antigen receptor T cells: Recognition and management. *Blood* **127**, 3321–3330 (2016).

35. L. A. K. Fonkoua, O. Sirpilla, R. Sakemura, E. L. Siegler, S. S. Kenderian, CAR T cell therapy and the tumor microenvironment: Current challenges and opportunities. *Mol. Ther. Oncolytics* **25**, 69–77 (2022).

36. T. Yan, L. Zhu, J. Chen, Current advances and challenges in CAR T-cell therapy for solid tumors: Tumor-associated antigens and the tumor microenvironment. *Exp. Hematol. Oncol.* **12**, 14 (2023).

37. V. M. Kadiri, C. Bussi, A. W. Holle, K. Son, H. Kwon, G. Schütz, M. G. Gutierrez, P. Fischer, Biocompatible magnetic micro- and nanodevices: Fabrication of FePt nanopropellers and cell transfection. *Adv. Mater.* **32**, 2001114 (2020).

38. F. Qiu, S. Fujita, R. Mhanna, L. Zhang, B. R. Simona, B. J. Nelson, Magnetic helical microswimmers functionalized with lipoplexes for targeted gene delivery. *Adv. Funct. Mater.* **25**, 1666–1671 (2015).

39. T. Bagci-Onder, H. Wakimoto, M. Anderegg, C. Cameron, K. Shah, A dual PI3K/mTOR inhibitor, PI-103, cooperates with stem cell-delivered TRAIL in experimental glioma models. *Cancer Res.* **71**, 154–163 (2011).

40. P. Erkoc, A. Cingöz, T. Bagci-Onder, S. Kizilel, Quinacrine mediated sensitization of glioblastoma (GBM) cells to TRAIL through MMP-sensitive PEG hydrogel carriers. *Macromol. Biosci.* **17**, 1600267 (2017).

41. H. Walczak, M. A. Degli-Esposti, R. S. Johnson, P. J. Smolak, J. Y. Waugh, N. Boiani, M. S. Timour, M. J. Gerhart, K. A. Schooley, C. A. Smith, R. G. Goodwin, C. T. Rauch, TRAIL-R2: A novel apoptosis-mediating receptor for TRAIL. *EMBO J.* **16**, 5386–5397 (1997).

42. J. M. Pimentel, J.-Y. Zhou, G. S. Wu, The role of TRAIL in apoptosis and immunosurveillance in cancer. *Cancer* **15**, 2752 (2023).

43. A. Montinaro, H. Walczak, Harnessing TRAIL-induced cell death for cancer therapy: A long walk with thrilling discoveries. *Cell Death Differ.* **30**, 237–249 (2023).

44. X. Qiao, S. Guo, Z. Meng, H. Gan, Z. Wu, Y. Sun, S. Liu, G. Dou, R. Gu, Advances in the study of death receptor 5. *Front. Pharmacol.* **16**, 1549808 (2025).

45. U. Anand, A. Dey, A. K. S. Chandel, R. Sanyal, A. Mishra, D. K. Pandey, V. De Falco, A. Upadhyay, R. Kandimalla, A. Chaudhary, J. K. Dhanjal, S. Dewanjee, J. Vallamkondu, J. M. Pérez de la Lastra, Cancer chemotherapy and beyond: Current status, drug candidates, associated risks and progress in targeted therapeutics. *Genes Dis.* **10**, 1367–1401 (2023).

46. X. Yuan, A. Gajan, Q. Chu, H. Xiong, K. Wu, G. S. Wu, Developing TRAIL/TRAIL death receptor-based cancer therapies. *Cancer Metastasis Rev.* **37**, 733–748 (2018).

47. M. R. Loebinger, A. Eddaoudi, D. Davies, S. M. Janes, Mesenchymal stem cell delivery of TRAIL can eliminate metastatic cancer. *Cancer Res.* **69**, 4134–4142 (2009).

48. M. R. Soboleski, J. Oaks, W. P. Halford, Green fluorescent protein is a quantitative reporter of gene expression in individual eukaryotic cells. *FASEB J.* **19**, 440 (2005).

49. E. M. Materón, C. M. Miyazaki, O. Carr, N. Joshi, P. H. Picciani, C. J. Dalmaschio, F. Davis, F. M. Shimizu, Magnetic nanoparticles in biomedical applications: A review. *Appl. Surf. Sci. Adv.* **6**, 100163 (2021).

50. S.-W. Chou, Y.-H. Shau, P.-C. Wu, Y.-S. Yang, D.-B. Shieh, C.-C. Chen, In vitro and in vivo studies of FePt nanoparticles for dual modal CT/MRI molecular imaging. *J. Am. Chem. Soc.* **132**, 13270–13278 (2010).

51. H. Shen, S. Cai, Z. Wang, Z. Ge, W. Yang, Magnetically driven microrobots: Recent progress and future development. *Mater. Des.* **227**, 111735 (2023).

52. A. Hahn, J. Fuhlrott, A. Loos, S. Barcikowski, Cytotoxicity and ion release of alloy nanoparticles. *J. Nanopart. Res.* **14**, 686 (2012).

53. C. M. Oral, M. Pumera, In vivo applications of micro/nanorobots. *Nanoscale* **15**, 8491–8507 (2023).

54. Z. X. Chong, S. K. Yeap, W. Y. Ho, Transfection types, methods and strategies: A technical review. *PeerJ* **9**, e11165 (2021).

55. R. Hannah, M. Beck, R. Moravec, T. Riss, CellTiter-Glo™ luminescent cell viability assay: A sensitive and rapid method for determining cell viability. *Promega Cell Notes* **2**, (2001).

56. M. Ghasemi, T. Turnbull, S. Sebastian, I. Kempson, The MTT assay: Utility, limitations, pitfalls, and interpretation in bulk and single-cell analysis. *Int. J. Mol. Sci.* **22**, 12827 (2021).

57. P. R. Cullis, M. J. Hope, Lipid nanoparticle systems for enabling gene therapies. *Mol. Ther.* **25**, 1467–1475 (2017).

58. N. O. Dogan, U. Bozuyuk, P. Erkoc, A. C. Karacakol, A. Cingoz, F. Seker-Polat, M. A. Nazeer, M. Sitti, T. Bagci-Onder, S. Kizilel, Parameters influencing gene delivery efficiency of PEGylated chitosan nanoparticles: Experimental and modeling approach. *Adv. NanoBiomed Res.* **2**, 2100033 (2022).

59. A. S. Arnold, V. Laporte, S. Dumont, A. Appert-Collin, P. Erbacher, G. Coupin, R. Levy, P. Poindron, J. P. Gies, Comparing reagents for efficient transfection of human primary myoblasts: FuGENE 6, Effectene and ExGen 500. *Fundam. Clin. Pharmacol.* **20**, 81–89 (2006).

60. J. Yoo, Y. Jung, K. Char, Y. Jang, Advances in cell coculture membranes recapitulating in vivo microenvironments. *Trends Biotechnol.* **41**, 214–227 (2023).

61. R. Großmann, I. S. Aranson, F. Peruani, A particle-field approach bridges phase separation and collective motion in active matter. *Nat. Commun.* **11**, 5365 (2020).

62. A. W. Mahoney, N. D. Nelson, K. E. Peyer, B. J. Nelson, J. J. Abbott, Behavior of rotating magnetic microrobots above the step-out frequency with application to control of multi-microrobot systems. *Appl. Phys. Lett.* **104**, 144101 (2014).

63. P. Guerrero-López, A. Martín-Pardillos, J. Bonet-Aleta, A. Mosseri, J. L. Hueso, J. Santamaria, J. M. Garcia-Aznar, 2D versus 3D tumor-on-chip models to study the impact of tumor organization on metabolic patterns in vitro. *Sci. Rep.* **15**, 19506 (2025).

64. H. E. Talbott, S. Mascharak, M. Griffin, D. C. Wan, M. T. Longaker, Wound healing, fibroblast heterogeneity, and fibrosis. *Cell Stem Cell* **29**, 1161–1180 (2022).

65. M. A. Kisiel, A. S. Klar, "Isolation and culture of human dermal fibroblasts," in *Skin Tissue Engineering: Methods and Protocols* (Springer, 2019), pp. 71–78.

66. Y. Liu, P.-C. Wu, S. Guo, P.-T. Chou, C. Deng, S.-W. Chou, Z. Yuan, T.-M. Liu, Low-toxicity FePt nanoparticles for the targeted and enhanced diagnosis of breast tumors using few centimeters deep whole-body photoacoustic imaging. *Photoacoustics* **19**, 100179 (2020).

67. I. C. Kurt, I. Sur, E. Kaya, A. Cingoz, S. Kazancioglu, Z. Kahya, O. D. Toparlak, F. Senbabaoglu, Z. Kaya, E. Ozyerli, S. Karahuseyinoglu, N. A. Lack, Z. H. Gumus, T. T. Onder, T. Bagci-Onder, KDM2B, an H3K36-specific demethylase, regulates apoptotic response of GBM cells to TRAIL. *Cell Death Dis.* **8**, e2897 (2017).

68. M. M. Salek, P. Sattari, R. J. Martinuzzi, Analysis of fluid flow and wall shear stress patterns inside partially filled agitated culture well plates. *Ann. Biomed. Eng.* **40**, 707–728 (2012).

69. L. Goto-Silva, N. M. Ayad, I. L. Herzog, N. P. Silva, B. Lamien, H. R. Orlande, A. da Costa Souza, S. Ribeiro, M. Martins, G. B. Domont, M. Junqueira, F. Tovar-Moll, S. K. Rehen, Computational fluid dynamic analysis of physical forces playing a role in brain organoid cultures in two different multiplex platforms. *BMC Dev. Biol.* **19**, 3 (2019).

70. E. Roux, P. Bougaran, P. Dufourcq, T. Couffinhal, Fluid shear stress sensing by the endothelial layer. *Front. Physiol.* **11**, 861 (2020).

71. E. Ozyerli-Goknar, I. Sur-Erdem, F. Seker, A. Cingöz, A. Kayabolen, Z. Kahya-Yesil, F. Uyulur, M. Gezen, N. Tolay, B. Erman, M. Gonen, J. Dunford, U. Oppermann, T. Bagci-Onder, The fungal metabolite chaetocin is a sensitizer for pro-apoptotic therapies in glioblastoma. *Cell Death Dis.* **10**, 894 (2019).
